# Molecular dissection of quantitative variation in fiber elongation between *Gossypium hirsutum* and *Gossypium barbadense* in reciprocal near-isogenic lines

**DOI:** 10.3389/fpls.2025.1657140

**Published:** 2025-09-16

**Authors:** Jeevan Adhikari, Deepak Vitrakoti, Wiriyanat Ployaram, Sameer Khanal, Rahul Chandnani, Jinesh Patel, Tariq Shehzad, Peng Chee, Andrew H. Paterson

**Affiliations:** ^1^ Plant Genome Mapping Laboratory, University of Georgia, Athens, GA, United States; ^2^ Department of Crop and Soil Sciences, University of Georgia, Tifton, GA, United States

**Keywords:** near-isogenic lines (NILs), cotton, interspecific introgression, genotyping-by-sequencing (GBS), fiber quality traits

## Abstract

In reciprocal interspecific near-isogenic lines developed by crossing elite cultivars Acala Maxxa (*Gossypium hirsutum*) and Pima S6 (*G. barbadense*) representing the two major domesticated species of cotton, we identified genomic locations underpinning an important fiber quality trait - fiber elongation (ELO). Phenotypic evaluation of these lines in three environments revealed a total of 36 QTLs, including 14 (38.89%) on the D subgenome, from a progenitor that does not produce spinnable fiber. Nearly half (16, 44.4%) of the 36 QTLs identified in the study explained less than 6% of phenotypic variation, and two (EL07.1 and EL25.1) were new, justifying the use of near-isogenic lines for analysis. Significantly larger additive effects of these QTLs in comparison to those reported using early generation backcrosses, F_2_ and F_2_ derived populations as well as recombinant inbred lines (RILs) show that NILs offer an advantage in estimating more precise QTL effects by removing background noise due to segregating genomic regions. Seven genomic regions on chromosomes 2, 6, 9, 12, 15 and 18 were consistently associated with ELO in two of the three environments tested. A total of 11 (30.56% of) QTLs had transgressive allele effects, i.e. which were opposite of what would be predicted from the parental phenotypes, indicating opportunities to breed superior interspecific lines; and three QTLs (8.33%) had heterotic alleles that may contribute to the striking fiber quality of F_1_ hybrids between these species. Limited reciprocity of QTLs in the two backgrounds is attributed to the combined consequences of epistasis, small phenotypic effects and imperfect coverage of donor chromatin in the recipient background. The availability of DNA markers linked to both *G. barbadense* and *G. hirsutum* QTLs identified in this and other studies promise to assist breeders in transferring and maintaining valuable traits from exotic sources during cultivar development.

## 1 Introduction

Cotton is an economically important crop and a key source of natural fibers for the textile industry. While consumer preferences and technological advances demand improved cotton fibers, genetic impoverishment of elite gene pools due to a series of bottlenecks imposed by polyploid formation, domestication and selection has been a hindrance to improvement (Adhikari et al., 2017; Paterson et al., 2004). Growing concerns about genetic vulnerability in many crop species including cotton has stimulated interest in utilizing secondary or tertiary gene pools as source of genetic variation in breeding programs. Closely related species often contain novel alleles, a subset of which might have potential for crop improvement. However, utilization of a close species can be difficult as the genetic variation between the species is often directly related to reproductive isolation, or to adaptation to different natural environments. Moreover, many introgressed genes prove to be difficult to use in crop improvement, particularly due to segregation distortion (Jiang et al., 2000), suppression of recombination (Paterson et al., 1990) and linkage drag (Young and Tanksley, 1989).

Cotton fiber production is dominated by two tetraploid species that are each cultivated for somewhat different characteristics. Upland cotton (*G. hirsutum*) is the most widely grown cotton because of its high-yield potential as well as adaptation to diverse environmental conditions and production systems. Pima, Egyptian and Sea Island cottons (all primarily *G. barbadense*, with introgression in specific chromosomal regions from *G. hirsutum* – (Wang et al., 1995) have superior fiber quality but lower yield potential and narrow environmental adaptation than Upland cotton. For example, Pima cotton’s narrow range of adaptation – primarily irrigated regions in arid zones of Arizona and California – precludes its commercial cultivation in most of the US “cotton belt”.

The superior fiber properties of *G. barbadense* have long been attractive for transfer to higher-yielding Upland cotton, a long-standing goal of cotton breeders and geneticists. DNA markers provide a means to detect and resolve complications such as segregation distortion or linkage drag encountered during interspecific gene introgression (Chee et al., 2005a). In particular, the advanced backcross approach facilitates the detection and integration of beneficial QTLs from secondary gene pools into elite breeding lines (Tanksley and Nelson, 1996). Elevated noise from multiple chromosomal segments segregating independently, some of which span entire chromosomes, often results in over- or underestimation of the effects of mapped QTLs and thus in lack of reproducible effects across environments and/or generations. Experimental populations developed by advanced backcross approaches consist of lines that have a few (in some cases only one) small introgressed segments segregating in the recipient background. These genetic stocks with reduced background noise and smaller size of introgressed chromatin segments facilitate estimation of the location and effects of these genomic benchmarks more precisely.

Advanced backcross lines (ABLs) with few introgressed chromosome segments from a donor and/or near-isogenic lines (NILs) with only one, are important genetic resources to estimate precise locations and accurate effects of quantitative trait loci (QTLs). The simple genomic composition of these lines serves two important purposes, both delineating the boundaries of a QTL to a small and precise genomic location and increasing the ability to detect QTLs of small phenotypic effects by reducing extraneous genetic variance at other loci and therefore reducing total phenotypic variance. Indeed, since there are few (ideally one) introgressed segment(s) in each line, phenotypes due to QTLs on the segment(s) often resemble simple Mendelian factors (Paran and Zamir, 2003). In addition, because of the fixed genotype of NILs, they can be replicated in different environments to test interaction between genetic and environmental factors (Monforte and Tanksley, 2000). By crossing NILs to the recurrent parent and obtaining recombinants in the introgressed region, fine mapping of specific QTLs toward their cloning is facilitated.

While long and fine cotton fibers were sought by the textile industry in the early years of mechanization, mostly due to their direct impact on the yarn, spinnability and end products (Perkins et al., 1984), other fiber properties eventually were found to contribute to overall textile performance, both in the textile mills as well as to the consumers. Fiber elongation, which measures the degree of extensibility or elasticity of the fibers before a break occurs, is becoming increasingly important as increases in elongation are associated with improved yarn strength (Riley, 1997). Modern textile mills are adopting sophisticated and more efficient spinning technologies that rely on high-speed and automation to achieve higher performances. Therefore, fibers with good elongation generally cause less costly disruption in the spinning process and the resulting yarn can endure more vigorous mechanical handing during fabric manufacturing.

In this study we are exploring the genetic architecture of important fiber quality parameters in a set of reciprocal interspecific advanced backcross populations (ABLs and NILs), using Acala Maxxa (*G. hirsutum*) and Pima S6 (*G. barbadense*) as parents. The respective populations tile 71.48% of the Acala Maxxa genome in Pima S6 (hereafter GB) background; and 78.72% of the Pima S6 genome in the Acala Maxxa (hereafter GH) background.

## 2 Materials and methods

### 2.1 Population development

Plant materials used in this study were developed from reciprocal crosses between *Gossypium hirsutum* acc. Acala Maxxa and *G. barbadense* acc. Pima S6 (both inbred lines). These genotypes have been extensively used to produce several molecular tools and resources including BAC libraries and Illumina genome sequences. Reciprocal advanced backcross populations were developed by first crossing the parents in a two-way cross (Acala Maxxa (♀) × Pima S6 (♂) – GH background; and Pima S6 (♀) × Acala Maxxa (♂) – GB background), then independently backcrossing F_1_ plants to the respective female parents to create 300 to 400 BC_1_ progenies for each cross. The backcrossing scheme utilized single seed descent to generate each subsequent generation (Adhikari et al., 2023).

After five generation of backcrossing, 179 BC_5_F_1_ plants from the GH background and 190 BC_5_F_1_ plants from the GB background were self-pollinated and a total of 369 BC_5_F_2_ families were grown at Iron Horse Farm (IHF), Watkinsville, Georgia in 2019 and 2021 and at Southwest Georgia Research Station, Plains, Georgia in 2021 under cultural conditions consistent with commercial irrigated cotton production. Individual BC_5_F_2_ plants that contained only one introgressed segment from the donor parent were deemed as NILs. Selfed seeds of these NILs (BC_5_F_3_ seeds) were grown at IHF and Plains in 2021 under cultural conditions consistent with commercial irrigated cotton production.

### 2.2 Genotyping

The genomic composition of the BC_5_F_1_ plants was inferred based on genotyping by sequencing (GBS). DNA was extracted from the parents and 369 BC_5_F_1_ plants using a scaled-down version of a published CTAB protocol (Paterson et al., 1993). A total of five multiplexed GBS libraries were constructed according to (Andolfatto et al., 2011) wherein the DNA were double digested with H*in*P1I-H*ae*III enzymes. The libraries were sequenced on Illumina MiSeq (in-house) with 75 bp single end reads (SE75). The TASSEL5 GBSV2 pipeline was used for sequence data processing and genotype calling (Glaubitz et al., 2014). Reads were aligned to *G. hirsutum* acc. *TM-1* (Zhang et al., 2015) using Burrow-Wheeler Alignment (bwa) and exported to variant call format (VCF). To minimize sequencing errors, only the first 64 base pairs were used to map reads to the reference genome. Filtering of the VCF was done for bi-allelic SNPs using Fisher’s exact test with a threshold *P* value < 0.001, considering that true variants should represent biallelic homozygous state for inbred accessions. Genotypes for lines in *G. hirsutum* background were called together with one another; and those for lines in *G. barbadense* background cross were also called together. The SNPs were filtered for MAF > 0.01, missing scores<30% and heterozygosity<10% at the population level. The retained SNPs were imputed using the Fast Inbred Line Library Imputation (FILLIN) pipeline available in TASSEL5 GBSv2 (Swarts et al., 2014).

The genomic composition of BC_5_F_2_ plants were inferred based on targeted microsatellite (SSR) genotyping of the introgressed chromosomal segments identified in their respective BC_5_F_1_ parents. At least two (and at most four) SSR markers were used to verify most of the introgressed regions while for small introgressions only one SSR marker was deployed. A total of 852 polymorphic SSR markers (**Supplementary Table 1**) spanning the introgressed regions were derived from several published genetic maps of crosses between *G. barbadense* and *G. hirsutum* stored in the CottonGen SSR database (https://www.cottongen.org/data/download/marker). A total of 47 candidate SSRs were monomorphic in our lines and discarded, as were 23 with ambiguous bands. Among the 8364 BC_5_F_2_ individuals planted in 2019, the remaining 782 SSR markers were used to genotype 5315 plants (from BC_5_F_1_ parents carrying 2 to 5 introgressions) for the presence (or absence) and dosage (homozygous vs heterozygous) of the respective introgression/s. Individual BC_5_F_2_ plants that were verified by SSR markers to carry only one introgression (homozygous or heterozygous) from the donor parent were deemed as NILs.

### 2.3 Phenotypic evaluation and data analysis

Two replications of each BC_5_F_2_ family and NIL were planted in a randomized complete block design (RCBD) in three environments (IHF-2019, IHF-2021 and Plains-2021). Six replications each of the two parents were included in all three environments. Fiber samples were collected by harvesting 25 bolls from each plot, ginned in a laboratory gin, and evaluated by HVI (Cotton Incorporated Textile Service Laboratory, Cary, NC) to extract values for fiber elongation (ELO).

All statistical analyses were conducted in R programming language. Single marker analyses were done in R/qtl (Broman and Sen, 2009). The significance threshold was set to LOD of 3, to mitigate the multiple-comparison problem. Filtration of significant markers adopts the method proposed by Szalma et al. (2007). If several markers on the same introgressed segment show significant association with phenotype, the most significant one was reported. For the co-segregation of multiple introgressions, the QTL location is examined as follows. First, if multiple families show significance for the trait and carry overlapping introgression, the introgression is considered to carry QTL. Second, if the co-segregation of introgressions is in single families, the most significant introgression is considered to carry QTL.

Phenotypic variance explained by each locus was reported by taking the most significant marker as independent variable and phenotypic value as dependent variable in R (R Core Team, 2016). Additive effects were estimated by half the difference of phenotypic values between the lines carrying the homozygous introgression and lines not carrying the introgression. Dominance effects were estimated by the difference of phenotypic values between the lines carrying the heterozygous introgression and the remaining lines that do not carry the introgression. If multiple or overlapping introgressions were present at both homozygous and heterozygous state, the estimation of additive effects utilized the lines carrying the introgression at homozygous state only and, the estimation of dominance utilized the lines carrying the introgression at heterozygous state only.

Gene actions for QTLs were determined by calculating the degree of dominance (absolute values) for every QTL that has both additive and dominance effects. The degree of dominance is the ratio of dominance effect to additive effect (d/a) of the QTL and based on this ratio, gene action of the QTLs can be categorized as (i) additive (0<d/a< 0.2) (ii) partially dominant (0.2< d/a<0.8) (iii) dominant (0.8< d/a< 1.2 and (iv) over-dominant (d/a > 1.2) (Edwards et al., 1987). QTLs with dominant and over-dominant effects are considered to have heterotic effect or heterozygous advantage.

### 2.4 Identification of common QTLs

‘Common QTL’ refers to cases in which the same marker is detected in two reciprocal populations, or two different markers are statistically significant for the same/overlapping QTL(s)/introgression(s) in each of two populations. The hypothesis that two sets of QTLs are randomly distributed across the entire genome can be falsified using the hypergeometric probability function (Paterson et al., 1995; Feltus et al., 2006). In the model, *p* is the probability of non-random correspondence of QTLs being compared; *n* is the number of comparable intervals which is calculated by dividing the total genome size by average introgression size in both populations; *m* is the number of common QTLs; *l* is the number of QTLs in the GH background; *s* is the number of QTLs in the GB background. The same model was also adopted to detect correspondence between QTLs reported in this study with those previously published. In this case, *l* is the total number of QTLs identified in the larger sample (study reporting higher number of QTLs) and *s* is the number of QTLs identified in the smaller sample.


$$p=\frac{\left(\begin{array}{c} l\\ m \end{array}\right)\left(\begin{array}{c} n-1\\ s-m \end{array}\right)}{\left(\begin{array}{c} n\\ s \end{array}\right)}$$

### 2.5 Candidate gene identification

*In silico* annotation was performed on the identified QTLs to look for candidate genes related to fiber elongation in cotton. For each QTL identified in the study, the genomic region spanning 1 Mb on each side of the most significantly associated marker was used for in silico analysis. The DNA sequence from this tightly linked region was used to look for *G. hirsutum* genes in the CottonGen database and these genes were then analyzed for biological functions, with particular focus on fiber growth and development.

## 3 Results

### 3.1 Genomic composition of NILs and ABLs

Detailed description of the genomic composition of the reciprocal advanced backcross lines (BC_5_F_1_ populations) is published (Adhikari et al., 2023, 2025). The NIL population in the Acala Maxxa (*G. hirsutum*) background consisted of 397 individuals with only one chromosomal segment introgressed from the donor parent (*G. barbadense* ‘Pima S6’), while that in the Pima S6 background consisted of 423 individuals. In total, these lines covered 78.72% and 71.48% of the donor genome respectively in the Acala Maxxa and Pima S6 backgrounds. Single introgressed segments were identified for all chromosomes except chromosome 24 in the Acala Maxxa background and chromosome 25 in the Pima S6 background. Individual lines contained an average of 0.904% of the donor genome, ranging from 0.07% (1.37 Mb) to 2.92% (56.65 Mb) in the Acala Maxxa background while in the Pima S6 background, individual lines contained an average of 0.97% of the donor genome, ranging from 0.15% to 3.07%.

### 3.2 Phenotypic performance of parents and experimental populations

**Figure 1:**
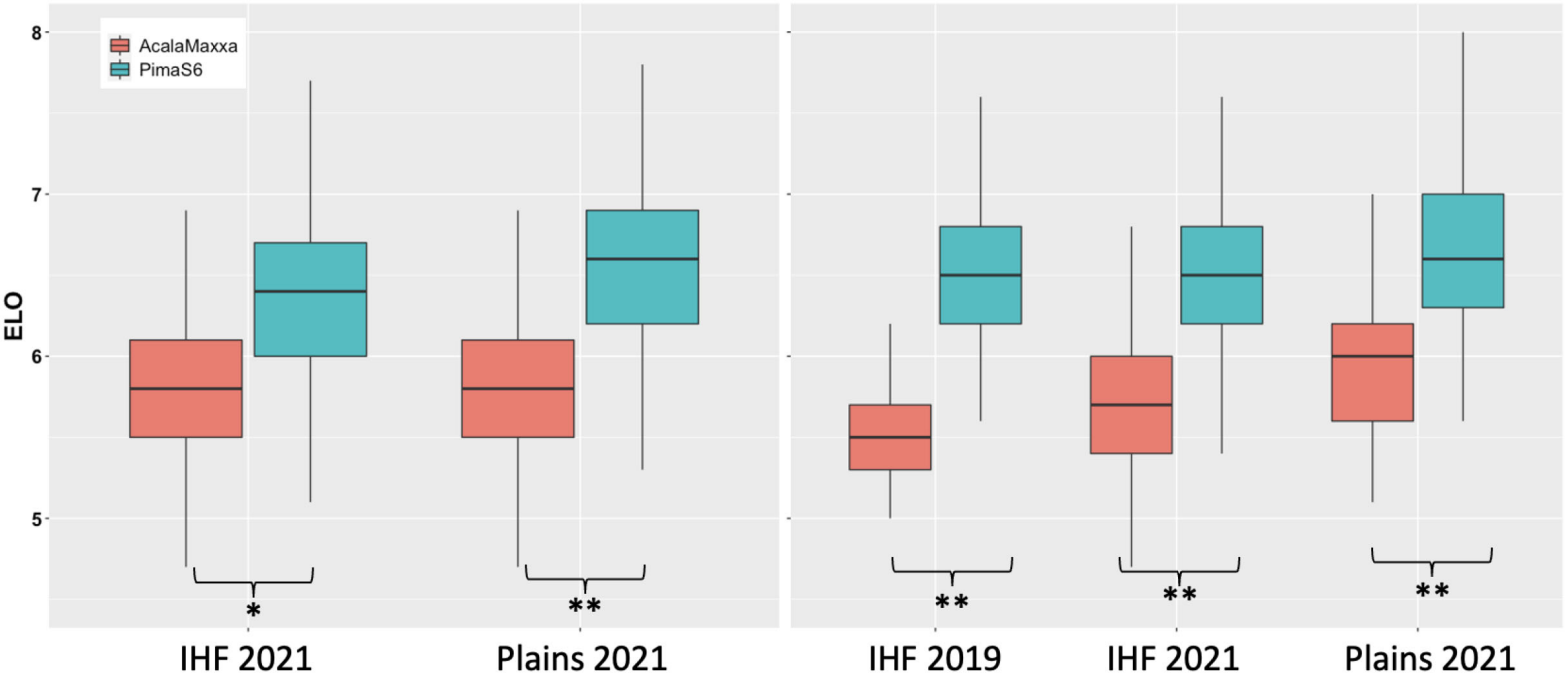
Distribution of fiber quality traits for the parents, reciprocal advanced backcrosses and NILs. Panels on the left show distribution of NILs for the two environments tested and panels on the right show distribution of the traits for advanced backcross populations. Significant at **0.01 and *0.05 alpha.

The phenotypic performance of the two parents, reciprocal backcross populations, and reciprocal NIL populations for fiber elongation is shown in **Figure 1**. The distribution of fiber elongation was approximately normal (Shapiro and Wilk test; p > 0.05) and typical of quantitative inheritance. Pima S6 outperformed Acala Maxxa in all three environments (**Supplementary Tables 2**, **3**; **Figure 1**). Both advanced backcross and NIL populations in the GB background performed better than in the GH background. Transgressive segregation is seen for all populations across all environments tested. Transgressive segregants outperforming both parents were identified in both population types and both backgrounds (**Figure 1**).

To identify the effect of genotypes and environment in the overall performance of the advanced backcross populations and the NILs, we conducted analysis of variance (ANOVA) treating all variables as fixed factors. Results showed significant effects of both genotype (GEN), and genotype-by-environment (GXE) (**Supplementary Table 4**). GEN captured the most variation in both population types and GXE also captured significant variation, precluding the use of combined phenotypic values in identification of QTLs. Thus, marker-trait association and identification of fiber elongation QTLs was performed separately for each environment tested.

### 3.3 Marker-trait association and overview of QTLs

A total of 36 marker-trait associations were identified for fiber elongation. Phenotypic variances explained by these QTLs ranged from 3.01% to 18.41% (**Tables 1**, **2**). Among the 36 QTLs identified, 11 were of large effect, explaining > 10% of the total phenotypic variation while the remaining 25 were small effect QTLs (explaining<10% of total phenotypic variation).

**Table 1:** QTLs for fiber elongation identified in the Acala Maxxa background.

QTL Name^#^	ENV	Marker*	LOD	PVE	a	d	d/a
EL01.1	IHF 2021	S1_85772418	3.54	4.35	0.46		
EL02.1	IHF 2021	S2_8696312	3.22	4.13	1.87	-0.63	-0.34
EL02.2	Plains 2021	S2_8987426	5.35	6.51	2.19	1.29	0.59
EL03.1	IHF 2019	S3_4311227	5.13	17.39		1.33	
EL05.1	IHF 2021	S5_85261315	4.21	4.85	0.41	1.65	4.02
EL06.1	IHF 2021	S6_5856948	4.99	3.01	0.99		
EL09.1	Plains 2021	S9_7254005	6.29	7.61	1.89	-0.29	-0.15
EL10.2	IHF 2019	S10_98258409	3.61	12.81		0.55	
EL12.1^i^	IHF 2019	S12_15376394	5.55	18.41		0.71	
EL12.1^ii^	Plains 2021	S12_15376394	4.05	15.03		0.25	
EL12.2^i^	IHF 2019	S12_51105721	4.83	16.28		0.59	
EL12.2^ii^	IHF 2021	S12_51105721	3.66	15.53		0.51	
EL14.1	IHF 2021	S14_58546089	6.6	3.59	1.04		
EL15.1^i^	IHF 2019	S15_3094439	4.87	17.37		0.53	
EL15.1^ii^	Plains 2021	S15_3094439	4.26	5.18	0.88		
EL15.2	IHF 2019	S15_59647072	4.37	14.26		0.73	
EL20.1	IHF 2021	S20_53920551	8.62	4.15	0.61		
EL21.1	IHF 2021	S21_52944095	4.1	7.39		0.5	
EL23.1	IHF 2021	S23_57997331	4.34	9.82	0.37		
EL24.1	IHF 2019	S24_19787425	3.12	12.11		0.57	
EL25.1	IHF 2019	S25_16260844	3.53	13.35		0.56	

*indicates the most significant marker. ^#, i, ii^ in QTL names show we infer the same QTL to have been identified in two different environments. a, d and d/a represent additive, dominant and gene action for respective QTL.

**Table 2:** QTLs for fiber elongation identified in the Pima S6 background.

QTL Name	ENV	Marker*	LOD	PVE	a	d	d/a
EL03.2	IHF 2019	S3_84465939	4.54	12.03		0.61	
EL04.1	Plains 2021	S4_47743671	3.46	5.08	0.96	-0.45	-0.47
EL06.2^i^	IHF 2021	S6_14751779	4.42	6.35	-1.06	-0.51	0.48
EL06.2^ii^	Plains 2021	S6_14751779	3.14	4.63	-0.94	-0.48	0.51
EL07.1	IHF 2021	S7_39864766	3.97	5.59	1.22	1.82	1.49
EL08.1	IHF 2019	S8_39417905	4.72	7.39		-0.39	
EL08.2	IHF 2021	S8_66072956	3.1	3.67	-0.57	-0.33	0.58
EL09.2^i^	Plains 2021	S9_50755816	3.22	4.37	1.19	0.59	0.5
EL09.2^ii^	IHF 2021	S9_68857432	3.8	4.48	0.92	0.31	0.34
EL10.1	IHF 2021	S10_71211391	3.6	5.08	-0.37	2.02	-5.46
EL16.1	IHF 2021	S16_30879676	4.84	9.87		1.67	
EL18.1^i^	IHF 2021	S18_20136056	3.27	4.98	0.92	0.12	0.13
EL18.2^ii^	Plains 2021	S18_20136056	3.9	3.95	1.16	0.39	0.34
EL22.1	Plains 2021	S22_8187981	4.15	6.84		1.66	
EL26.1	Plains 2021	S26_8664132	3.81	6.09		0.89	

*indicates the most significant marker. ^#, i, ii^ in QTL names show the same QTL was identified in two different environments. a, d and d/a represent additive, dominant and gene action for respective QTL.

In the GH background, a total of 21 QTLs were identified for ELO in the three environments tested, explaining 3.01% to 18.41% of total phenotypic variation (**Table 1**). These QTLs were distributed over 15 chromosomes, with chromosome 12 carrying the most QTLs. A total of 11 of these QTLs were small effect and the remaining 10 were large effect. Among the 21 QTLs identified, eight were identified at IHF in 2019, nine at IHF in 2021 and four at Plains in 2021. All 21 QTLs identified in the GH background increased fiber elongation consistent with the parental phenotypes. The ELO QTL on chromosome 12 was identified in all three environments tested and is a potential candidate for fine mapping and QTL identification studies. Another QTL on chromosome 15 was identified at IHF in 2019 and at Plains in 2021, making it a priority for further study of its effect on fiber elongation.

In the GB background, a total of 15 QTLs were identified for ELO, explaining 3.67% to 12.03% of the total phenotypic variation (**Table 2**). Among the 15 QTLs identified for ELO, two were identified at IHF in 2019, seven at IHF in 2021 and six at Plains in 2021. Of the 15 QTLs, only on chromosome 3 was a major effect QTL and the remaining 14 were small effect QTLs. Five of these QTLs decreased fiber elongation while 10 increased fiber elongation. One QTL on chromosome 6 and one on chromosome 9 were each identified in two environments (IHF 2021 and Plains 2021).

### 3.4 Subgenomic distribution of QTLs

Among the 36 QTLs identified for fiber elongation, 22 were identified in the At subgenome and 14 were identified in the Dt subgenome. In both backgrounds, At subgenome carried more QTLs for fiber elongation than Dt subgenome (12 vs 10 in the GH background and 9 vs 5 in the GB background). QTLs for fiber elongation were identified in all chromosomes except chromosomes 11, 13 and 19. In the GH background, fiber elongation QTLs were identified in 15 of 26 chromosomes while in the GB background, they were identified in 11 chromosomes.

### 3.5 Transgressive and heterotic QTLs

A total of 11 QTLs showed transgressive effects for fiber elongation, i.e. which were opposite of what would be predicted by comparison to the parental phenotypes (**Table 3**). Five of the 11 transgressive QTLs were identified in the Acala Maxxa background while six were identified in the Pima S6 background. Except for EL08.1 which showed fiber elongation values lower than both the parents, all other transgressive QTLs outperformed the parents in both backgrounds. Two QTLs in Acala Maxxa background and one in Pima S6 background had average fiber elongation values above 7.0, far exceeding the performance of the better parent (Pima S6), thus, indicating the potential for their use in breeding superior cultivars.

**Table 3:** Transgressive QTLs identified in the study.

QTL Name^#^	ENV	Marker*	Average performance	Parental Average	
				Acala Maxxa	Pima S6
EL01.1	IHF 2021	S1_85772418	6.5 (5)	5.44	6.40
EL02.1	IHF 2021	S2_8696312	7.21 (1)	5.44	6.40
EL02.2	Plains 2021	S2_8987426	7.52 (1)	5.55	6.42
EL14.1	IHF 2021	S14_58546089	6.7 (3)	5.44	6.40
EL15.1^ii^	Plains 2021	S15_3094439	6.55 (5)	5.55	6.42
EL04.1	Plains 2021	S4_47743671	6.61 (6)	5.55	6.42
EL07.1	IHF 2021	S7_39864766	6.91 (2)	5.44	6.40
EL08.2	IHF 2021	S8_66072956	5.08 (9)	5.44	6.40
EL09.2^i^	Plains 2021	S9_50755816	7.12 (6)	5.55	6.42
EL09.2^ii^	IHF 2021	S9_68857432	6.9 (6)	5.44	6.40
EL18.2^ii^	Plains 2021	S18_20136056	6.65 (2)	5.55	6.42

The average performance in the table indicates the mean values of fiber elongation for lines carrying the respective QTL and the values in parenthesis indicate the number of lines carrying the QTL.

Among the 36 QTLs identified in the study, three showed heterotic effects (d/a >|1.2|, see methods section for details). All three QTLs showed overdominant gene effects (d/a: 1.49 for EL07.1, d/a: 4.02 for EL05.1 and d/a: -5.46 for EL10.1; **Tables 1**, **2**). It is well known that the F1 hybrids between these two species have striking fiber quality, and are directly used as cultivars in regions such as India where hybrid seed production costs are low. Further evaluation of salient lines carrying alleles such as these to validate their effects in multi-environment and multi-location trials may elucidate the genetic underpinnings of such hybrid advantages.

## 4 Discussion

Introgressive breeding approaches not only introduce novel allelic variation into cultivated gene pools, but interspecific populations developed using these approaches can be valuable for molecular dissection of complex fiber yield and quality parameters. Many past studies have focused on investigating the effects of GB chromatin segments introgressed into GH, however, the reverse has not been routinely studied. In the current study, we developed a reciprocal set of advanced backcross lines and NILs selected from among the selfed progenies of these advanced backcross lines and tested these populations to assess the effects of reciprocal chromatin transfer on an important fiber quality trait – ELO (fiber elongation). We identified a total of 36 marker-trait associations for fiber elongation with variable genetic effects. This study adds to the resources and observations available to study the quantitative nature of fiber quality traits reciprocally in two elite cotton backgrounds.

### 4.1 Performance of NILs and advanced backcross populations

NILs and advanced backcross populations in both backgrounds showed average phenotypes consistent with their recurrent parent (**Figure 1**). Albeit the average performance of these two populations behaved like their recurrent parents, which is expected given the exceptionally large proportion of their genome coming from the recurrent parent, a large amount of variation was observable (Adhikari et al., 2023, 2025). The presence of transgressive segregants in both directions suggests that the chromatin segments introgressed from the donor parents have effects that could significantly alter the performance of individual lines.

### 4.2 Effect of species background

The total number of QTLs identified for the three fiber quality traits differed little in the two backgrounds (21 in GH vs 15 in GB). While identification of similar total numbers of QTLs in the reciprocal backgrounds might suggest the involvement of similar number of genes in controlling these traits, many other findings indicate the effect of species background on effects, locations, and patterns of these QTLs. For example, all 21 QTLs identified in the GH background had positive effects, i.e. introgression from GB into GH always increased fiber elongation. While GB is known to have superior fiber elongation than GH and has the potential to transfer favorable alleles to GH background, it is an interesting observation to see that all the QTLs conferred by PimaS6 to the GH background increased fiber elongation. Observations like these, where alleles contributing favorable alleles are spread throughout the genome, would be suggestive of utilizing a comprehensive approach like genomic selection (rather than targeted selection) when breeding for such traits that have favorable alleles spread throughout the genome.

GH is usually not superior to GB in terms of fiber quality, and for this, it has rarely been used as a donor parent in trait mapping studies (Liu et al., 2023). The reciprocal nature of our populations allowed us not only to study GH as a recipient parent of favorable alleles from GB, but also to explore GH itself as potential source of novel alleles to the GB background. Our results suggest that GH can also contribute favorable alleles to GB. Of the 15 QTLs identified in the GB background, nine (more than half) had positive effects in increasing fiber elongation.

A perplexing observation, however, is the complete lack of reciprocity of QTLs identified with opposite phenotypic effects at corresponding locations in the reciprocal genetic backgrounds. In principle, one would expect the majority of QTLs to show such reciprocity if alternative alleles at a QTL show additive or dominant-recessive effects. Limited correspondence of identified QTLs in the two backgrounds could be a result of several factors. From first principles, noting that these lines covered 78.72% and 71.48% of the donor genome respectively in the Acala Maxxa and Pima S6 backgrounds, only a little more than half the genome is actually tested in both backgrounds. Further, the small phenotypic effects of most of the identified QTL, increases the likelihood that one or both members of a reciprocal pair elude detection (Broman, 2001). Another intriguing factor that could account for some failures to identify correspondence of QTLs in the reciprocal backgrounds, especially in advanced backcross lines with multiple introgressed chromosomal segments, is epistasis. Interaction between introgressed loci might result in underestimation of their effects which might have resulted in some QTLs failing to reach the biometric thresholds required to declare them as QTLs *per se*. The widespread observation that fiber quality parameters generally have high heritability (Fang et al., 2014) suggests a limited role of epistasis, but it could contribute to failures to identify reciprocal QTLs with relatively small effects (Chandnani et al., 2018). This might be the case here as most of the QTLs detected in this study show low genetic contribution to the total phenotypic variation explained by the phenotype.

### 4.3 Subgenomic distribution of fiber quality QTLs

While it is no longer controversial that an appreciable portion of variation in fiber quality derives from the D subgenome (Jiang et al., 1998) from an ancestor that does not produce spinnable fiber, the relative roles of the two cotton subgenomes vary by trait and population. With regard to the distribution of QTL in A and D subgenomes, many prior studies have concluded that more QTL for growing period, yield and fiber quality were distributed in the Dt subgenome than in the At subgenome (Rong et al., 2007; Said et al., 2013; Wang et al., 2013; Adhikari et al., 2017; Chandnani et al., 2018). However, Shen et al. (2005) and Lin et al. (2005) reported that more QTL for fiber quality were located in the At than the Dt subgenome. In the current study, we identified more QTLs in the At than the Dt subgenome. For NILs and advanced backcross populations in the GH background, the subgenomic affinities of QTLs were not significantly different (p>0.05) but nominally agreed with most previous findings, with the At subgenome harboring three more QTLs (12 vs 10) than the Dt subgenome. In the reciprocal background, however, a significantly higher (p<0.05) number of QTLs were identified in the At subgenome (9 vs 5).

### 4.4 Stability of fiber quality QTLs

**Figure 2:**
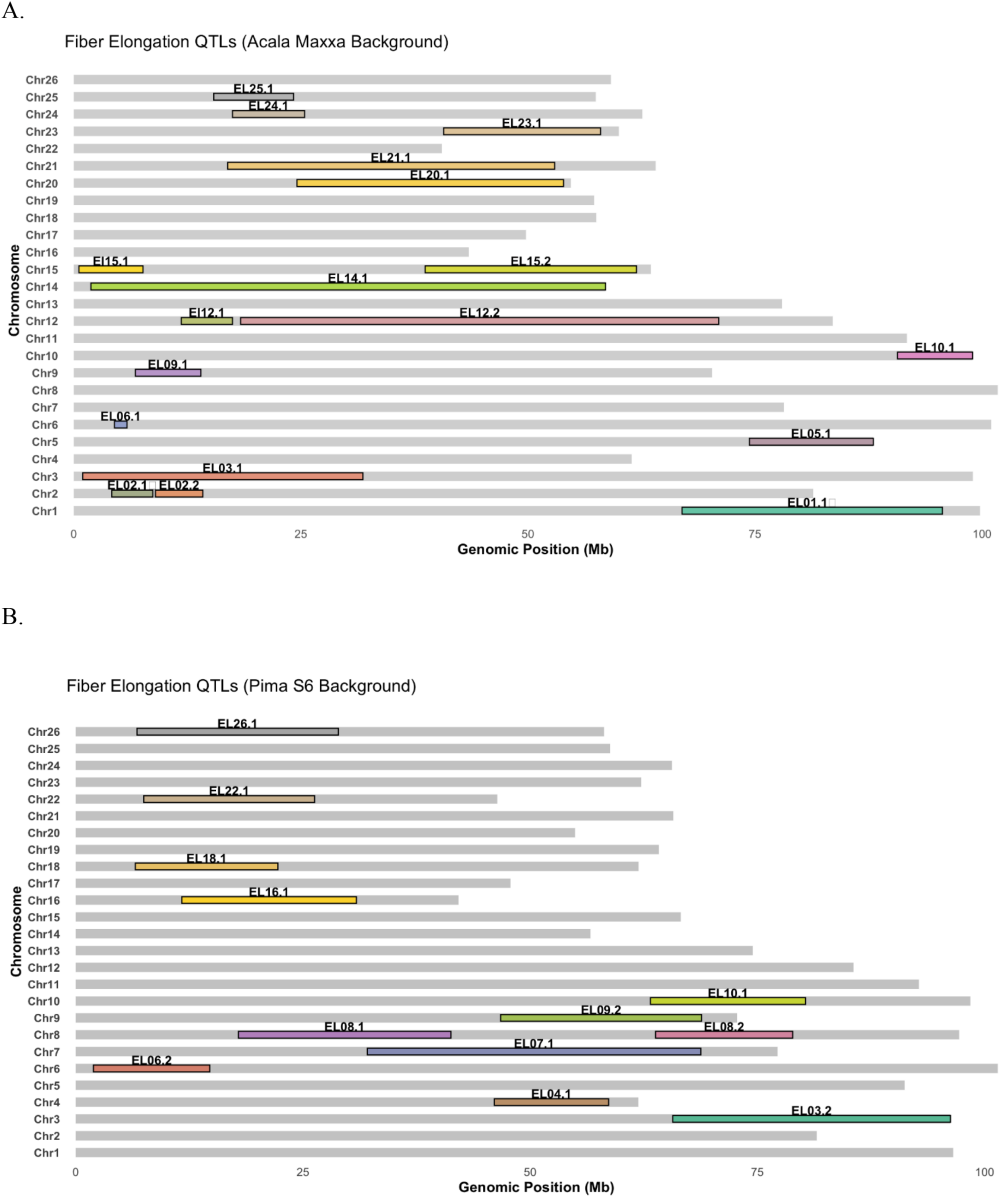
Distribution of QTLs associated with fiber elongation identified in **(A)** Acala Maxxa background **(B)** Pima S6 background.

While different loci associated with the same trait under various environments might suggest interaction between genotype and environment, QTLs being detected across environments might indicate environmental stability. Although most QTLs identified in this study were single-environment QTLs, seven genomic regions (on chromosomes 2, 6, 9, 12, 15 and 18) were consistently associated with ELO in two different environments (**Tables 1**, **2**, **Figure 2**). Since NILs facilitate pinpointing genomic regions affecting phenotypes to a rather small region around the causal mutation without accumulating a lot of extraneous variation, the identification of these seven stable regions for QTL in the cotton genome warrants more indepth look into these regions for genes controlling the trait. While this investigation is not in the scope of our current study, it will certainly help the scientific community to streamline efforts in these regions to identify and verify candidate genes controlling fiber elongation.

### 4.5 Similarity with QTLs previously reported

The genetic composition of our experimental populations provides a platform to identify novel small-effect QTLs in addition to major QTLs. Since NILs serve as a resource to not only identify marker trait associations but also an important tool to verify QTLs previously identified, mostly those using early generation populations, the correspondence identified here could be used as a means of validation of previously published QTLs. Given that there are hundreds of studies reporting fiber quality QTLs and owing to the genetic structure of our experimental populations, we mostly compared our results with populations of similar genomic composition (Chee et al., 2005b; Draye et al., 2005; Yu et al., 2013; Brown et al., 2019). We also performed elaborate statistical comparisons with other previous reports on the correspondence of QTLs identified in our study.

In a comprehensive study of biometric parameters of QTLs affecting fiber elongation using a backcross-self approach, (Chee et al., 2005a, b) identified a fiber elongation QTL (EL05.1) on chromosome 5 with the nearest locus being RFLP marker pAR206b, in the same introgressed segment where we identified the most significant marker for our ELO QTL (qEL05.1) in the GH background, both GB alleles increasing fiber elongation. In the same study, another fiber elongation QTL EL23.1 was identified on chromosome 23 with the nearest marker being an RFLP marker pAR209, near which we also identified a fiber elongation QTL (EL23.1) – albeit with a different phenotypic effect. While the fiber elongation QTLs identified on this chromosome in the previous study decreased fiber elongation, the one identified in our study increased fiber elongation. In both studies, the donor parent was the same (Pima S6) while the recipient parents were different (*G. hirsutum* cv. Tamcot 2111 in the previous study), implicating variation among cultivars within the same species as a possible cause of this difference.

While correspondence of individual marker-trait associations may reflect, for example, a gene for which the recurrent parent, Acala Maxxa or Pima S6, has a rare allele, non-random patterns of association across an entire genome can reflect other properties such as convergent domestication (Paterson et al., 1995). To investigate such genome wide non-random patterns of association, we also conducted a comprehensive study on the correspondence of QTLs identified here, with previous reports irrespective of the population type. Information on a total of 830 QTLs related to fiber elongation in 103 previous reports were downloaded from CottonGen (https://www.cottongen.org). The hypergeometric probability distribution was used to analyze QTL correspondence, providing a means to infer statistically whether QTLs for a trait are randomly distributed between two populations or environments. Correspondence was identified for 15 of the 31 previously reported studies for the QTLs reported in Acala Maxxa background and for 10 of the 31 previously reported studies in Pima S6 background (**Table 4**). Common QTLs were identified in most of the 31 reports listed, however, only 15 (or 10) significant P values based on the hypergeometric distribution suggest that across the genome, this correspondence is not sufficient to infer a non-random distribution of QTLs between published studies.

**Table 4:** Correspondence of fiber quality QTLs between current and previous study.

Previous Study			Common QTLs		P.value	
Pop	# QTLs	Reference	GH	GB	GH	GB
RIL	17	Liu et al., 2017	7	4	**0.001**	**0.023**
F2	35	Deng et al., 2019	7	4	**0.043**	0.157
F2:3	42	Diouf et al., 2018	7	6	0.088	**0.048**
F2	3	Huang et al., 2018	2	1	**0.031**	0.194
MAGIC	7	Huang et al., 2018	2	1	0.142	0.337
RIL	119	Jamshed et al., 2016	7	6	**0.004**	0.062
RIL	34	Jia et al, 2016	8	7	**0.013**	**0.004**
F2	6	Kumar et al., 2019	1	1	0.376	0.311
BC1	12	Lacape et al., 2005	5	4	**0.004**	**0.007**
RIL	22	Li et al., 2016	3	2	0.238	0.291
RIL	88	Li et al., 2018	11	6	0.149	0.204
F2	22	Liang et al., 2013	4	2	0.134	0.291
RIL	3	Liu et al., 2016	1	1	0.264	0.194
RIL	26	Ma et al., 2017	4	2	0.178	0.302
F2	5	Rong et al., 2007	3	3	**0.010**	**0.003**
RIL	10	Shang et al., 2015	4	1	**0.013**	0.384
RIL	50	Shang et al., 2015	7	4	0.144	0.234
F2:3	12	Shen et al., 2005	4	4	**0.026**	**0.007**
RIL	4	Shen et al., 2007	1	1	0.314	0.241
F2	10	Saranga et al., 2004	4	2	**0.013**	0.137
RIL	10	Tan et al., 2015	4	4	**0.013**	**0.003**
RIL	76	Tan et al., 2018	9	5	0.174	0.206
RIL	21	Tang et al., 2015	5	5	**0.046**	**0.010**
RIL	9	Wang et al., 2007	2	2	0.196	0.117
RIL	64	Wang et al., 2015	5	2	0.125	0.067
F2	67	Wang et al., 2016	7	5	0.188	0.223
BIL	12	Yu et al., 2013	5	2	**0.004**	0.174
RIL	12	Yu et al., 2014	4	3	**0.026**	**0.043**
RIL	3	Zhang et al., 2009	1	1	0.264	0.194
F2:3	2	Zhang et al., 2005	2	1	**0.012**	0.139
CSIL	27	Zhang et al., 2016	4	5	0.188	**0.028**

P-values obtained using hypergeometric probability distribution (see methods). Values in bold represent significant p-values, highlighting QTLs that had non-random correspondence with previously published reports.

### 4.6 Comparison of absolute effects with early generation populations

While it is a common practice to compare and contrast QTLs mapped in different populations to verify underlying loci, it is also important to highlight the advantages of some mapping populations over others, especially in terms of the quality of the estimates that they can offer. With reduced background noise, advanced backcross populations such as NILs offer more precise estimates of the effects of underlying QTLs. While the total variations attributable to the QTLs obtained from our NIL populations (**Tables 1**, **2**, **Figure 2**) are not as pronounced as those from F2, F2:3, RILs or other early generation populations reported elsewhere (which is expected given the nature of our populations), the effects of these QTLs individually were significantly larger than those reported by early generation studies of population types that carry a lot of segregating genomic regions (**Table 5**).

**Table 5:** Comparison of additive effects of fiber elongation QTLs identified using NILs (current study) versus early generation populations (published reports).

Population Type	# QTLs	min a	max a	ave a	t-test	Reference
F2, F2:3	9	0.02	0.32	0.13	**	DOI 10.1007/s11032-004-4731-0
RIL	4	0.11	0.15	0.12	**	DOI 10.1007/s10681-006-9224-2
RIL	2	0.11	0.15	0.11	**	DOI 10.1007/s10681-006-9338-6
RIL	11	0.02	0.09	0.05	***	DOI 10.1007/s10681-014-1189-y
BC1	6	0.08	0.17	0.12	**	DOI 10.1007/s10681-014-1194-1
BC	10	0.22	0.6	0.37	*	*CROP SCIENCE -MADISON-*. 45(1):123-140
F2	4	0.04	0.07	0.05	***	https://doi.org/10.1186/s42397-020-00076-y
F2	24	0.0003	0.023	0.009	***	https://doi.org/10.3390/agronomy14081719
F2	4	0.13	0.44	0.28	*	https://doi.org/10.1186/s12864-022-08528-2
Maxxa NILs	10	0.37	2.19	1.07		Current Study
Pima NILs	10	0.37	1.22	0.93		Current Study

minA, maxA and aveA represent the absolute values of the minimum, maximum and average additive effects reported in these studies. *, **, *** show deviation of reported means from the average effects reported here at 0.05, 0.01 and 0.001 levels of significance.

Since the parents used, type of crosses and population sizes, precision of reported location of the QTLs, and type of diagnostic markers for reporting QTLs in prior studies vary greatly, the distribution of the additive allele effects of the QTLs were compared to ours using a two-sample t-test. Absolute values of the effects were used to perform the t-test to remove/reduce cancellations of effect sizes due to the direction of the QTL source (sign convention). The QTLs reported in the current study have significantly larger average additive effects that those from other population types (**Table 5**). While, in general, fiber elongation QTLs from other population types contributed 0.009 to 0.37 percent additive effects with maximum observed values of 0.6 percent, the average additive effect of QTLs reported here average 1.07 percent with a maximum effect of 2.19 percent. Indeed, the minimum effect of 0.37 percent found in the NILs is greater than most averages or even maximums reported in other studies. These results suggest that NILs offer a much greater advantage in precisely identifying QTL effects, especially relatively subtle effects that might be masked by either genetic or non-genetic variation in other population structures.

### 4.7 *In silico* annotation of potential candidate genes

Availability of cotton reference genomes (Paterson et al., 2012; Zhang et al., 2015) enabled us to scrutinize physical regions surrounding the identified QTLs for genes/gene families known or suspected to affect fiber quality parameters in cotton. This investigation was limited to tightly linked regions i.e., 1 Mb on both sides of the SNP marker that is most significantly associated with the QTLs. On chromosome 5, the nearest gene of interest to an ELO QTL (EL05.1) was a *C2HC* zinc finger superfamily protein, a member of a gene family whose gene expression level at 11 days post-anthesis has been reported to be highly correlated with UHM and which has been implicated in secondary cell wall thickening (Al-Ghazi et al., 2009). Similarly, on chromosome 10, the nearest gene of interest to another fiber elongation QTL (EL10.2) was a glucosyltransferase like *UDP-Gp* gene. The enzymatic activity of this gene increases during the period of development when cotton fiber is synthesizing massive amount of cellulose (Taliercio and Kloth, 2004; Xu et al., 2022), which like protein is involved in cellulose biosynthesis during secondary cell wall formation stage. While it is premature to suggest the active roles of these genes towards fiber trait phenotypes, these genes are potential candidates for these roles given that they are so close to the most significant marker for fiber quality QTLs that have been repeatedly identified in multiple environments and studies. Improved genomic resources together with these novel populations are expected to accelerate candidate gene identification and validation for many such loci.

## 5 Conclusion

Breeding programs have often attempted the transfer of alleles between the two major species of cotton, with GB being the preferred donor due to its superior fiber quality parameters. A paucity of studies has been conducted on the reciprocal transfer primarily due to low fiber yield of GB and the hypothesis that GH may not contribute towards improving fiber quality of its sister species. Reciprocal experimental populations offer objective comparison of the potential of the two species not only in their contribution of favorable alleles to the recipient background but also in their permeability and/or resistance to alleles from the donor species.

By utilizing advanced reciprocal backcross lines and near-isogenic lines, we studied the impact of genetic background on chromatin transfer and their effect on a crucial fiber quality trait – fiber elongation. Not only were we able to show the effect of recipient background on this trait but we were also able to show that GH, the species with generally poorer fiber quality, nonetheless contributed favorable alleles at some loci for fiber elongation. The near-isogenic genomic composition of our population provided opportunities to estimate the effects of genomic regions more precisely, but at a cost of the ability to detect epistatic QTL interactions. With one of the major purposes of NILs being the ability to verify the location and effects of QTLs, in addition to their identification, we were able to validate important fiber quality QTLs identified in previous studies. Since the parents used in creating these populations are both elite lines representing the two major domesticated species of cotton, with their own specialties and differences, the populations we developed are not just a platform to identify genomic locations underpinning fiber quality traits, but also a good starting point from which to study divergence, domestication and evolutionary history of cotton as well as to select superior individual lines for breeding and/or commercialization.

## Data Availability

The raw SNPs data corresponding to the advanced backcross lines is available at https://doi.org/10.5061/dryad.kh189329v and the SSR marker data that have been used to validate the NILs is available as supplementary dataset (**Supplementary Table 1**).
