# Supplementary material for: Molecular dissection of quantitative variation in fiber elongation between Gossypium hirsutum and Gossypium barbadense in reciprocal near-isogenic lines
Source: Front Plant Sci. 2025 Sep 16;16:1657140. doi: 10.3389/fpls.2025.1657140 (PMC12479486; doi:10.3389/fpls.2025.1657140)
Supplement: Supplementary file 1 [file Table1.docx]

Supplementary Table 1. Summary of SSR markers used to verify the introgression in this study.

| Chr | At/Dt | Markers used for introgression verification |
| --- | --- | --- |
| 1 | A01 | NAU4045 (2245931-2246101) |
| 1 | A01 | HAU0699 (5512903-5513197) |
| 1 | A01 | NAU2419 (6258721-6258981) |
| 1 | A01 | NAU5100 (6665365-6665565) |
| 1 | A01 | BNL3580 (7078093-7078309) |
| 1 | A01 | NAU4073 (7954782-7954951) |
| 1 | A01 | BNL1667 (9407514-9407676) |
| 1 | A01 | BNL3090 (15352390-15352646) |
| 1 | A01 | DPL0094 (15436157-15436418) |
| 1 | A01 | TMB0062 (17562250-17562499) |
| 1 | A01 | DPL0887 (18148397-18148549) |
| 1 | A01 | MUSB0590 (21802173-21802503) |
| 1 | A01 | BNL2700 (22613414-22613564) |
| 1 | A01 | TMB2051 (23761366-23761548) |
| 1 | A01 | BNL3910 (24531795-24531993) |
| 1 | A01 | MUSB0818 (26242760-26243026) |
| 1 | A01 | TMB0585 (29505551-29505758) |
| 1 | A01 | BNL1418 (37811715-37811891) |
| 1 | A01 | BNL2921 (40133025-40133182) |
| 1 | A01 | NAU3384 (45168742-45168936) |
| 1 | A01 | NAU3385 (45168748-45168936) |
| 1 | A01 | DPL0615 (55444805-55445033) |
| 1 | A01 | MUSB0596 (59652815-59653189) |
| 1 | A01 | MUSB0059 (64565346-64565576) |
| 1 | A01 | MUSB0995 (66422249-66422519) |
| 1 | A01 | MUSB0749 (68273609-68273907) |
| 1 | A01 | DPL0110 (74970061-74970256) |
| 1 | A01 | BNL3652 (75424981-75425115) |
| 1 | A01 | NAU2095 (95794297-95794469) |
| 1 | A01 | MUCS164 (99687363-99687907) |
| 2 | A02 | JESPR0304 (66925-67069) |
| 2 | A02 | BNL2877 (517810-517923) |
| 2 | A02 | BNL3545 (927093-927280) |
| 2 | A02 | BNL3972 (2769132-2769370) |
| 2 | A02 | BNL1897 (5812291-5812433) |
| 2 | A02 | JESPR0293 (7292448-7292538) |
| 2 | A02 | BNL2651 (7944229-7944343) |
| 2 | A02 | MUSB1017 (8878757-8879083) |
| 2 | A02 | DPL0674 (11760322-11760555) |
| 2 | A02 | CIR0401 (12747425-12747550) |
| 2 | A02 | TMB1513 (13401735-13401969) |
| 2 | A02 | BNL3491 (13442232-13442788) |
| 2 | A02 | TMB0923 (13953776-13953983) |
| 2 | A02 | BNL2635 (15295312-15295473) |
| 2 | A02 | MUSB1220 (15722379-15722623) |
| 2 | A02 | MUSB0550 (22539638-22539957) |
| 2 | A02 | BNL3523 (24316875-24317071) |
| 2 | A02 | MUSB0309 (24942840-24942995) |
| 2 | A02 | HAU2923 (25142041-25142304) |
| 2 | A02 | DPL0261 (27382613-27382769) |
| 2 | A02 | MUSB0799 (27468945-27469227) |
| 2 | A02 | BNL3590 (40971023-40971204) |
| 2 | A02 | MUSS0599 (43561753-43562007) |
| 2 | A02 | BNL2706 (46474803-46475026) |
| 2 | A02 | NAU2817 (48254281-48254697) |
| 2 | A02 | MUSB0194 (48507013-48507188) |
| 2 | A02 | MUSB0799 (56721225-56721511) |
| 2 | A02 | MUSB0564 (56721851-56722290) |
| 2 | A02 | DPL0216 (60176821-60177049) |
| 2 | A02 | MUCS106 (60836062-60836269) |
| 2 | A02 | BNL3292 (62024994-62025109) |
| 2 | A02 | MUSB0888 (70167497-70167846) |
| 2 | A02 | BNL3015 (70741303-70741507) |
| 2 | A02 | CIR0251 (73325395-73325582) |
| 2 | A02 | DPL0073 (75174339-75174540) |
| 2 | A02 | NAU2325 (75587719-75587918) |
| 2 | A02 | HAU0880 (80045222-80045391) |
| 3 | A03 | TMB1540 (1009544-1009761) |
| 3 | A03 | BNL3408 (2815443-2815573) |
| 3 | A03 | DPL0045 (3969833-3970036) |
| 3 | A03 | BNL1606 (3969933-3970109) |
| 3 | A03 | DPL0529 (7332303-7332462) |
| 3 | A03 | NAU3427 (8827744-8827928) |
| 3 | A03 | BNL1379 (11649762-11650104) |
| 3 | A03 | HAU1022 (12620243-12620521) |
| 3 | A03 | BNL3441 (18355732-18355941) |
| 3 | A03 | NAU3808 (21279859-21280085) |
| 3 | A03 | NAU3639 (21636232-21636397) |
| 3 | A03 | DPL0901 (28166258-28166458) |
| 3 | A03 | BNL3267 (33473427-33473584) |
| 3 | A03 | MUSB0059 (36977126-36977356) |
| 3 | A03 | BNL3445 (41974144-41974675) |
| 3 | A03 | DPL0546 (42037298-42037504) |
| 3 | A03 | MUSB0611 (48867579-48867861) |
| 3 | A03 | DPL0734 (51660159-51660371) |
| 3 | A03 | MUSB0550 (53869081-53869390) |
| 3 | A03 | NAU1179 (55058690-55058920) |
| 3 | A03 | BNL1059 (55060824-55061030) |
| 3 | A03 | MUSS0192 (57158030-57158337) |
| 3 | A03 | NAU884 (57158142-57158340) |
| 3 | A03 | NAU3120 (62147639-62147846) |
| 3 | A03 | BNL3398 (70732628-70732826) |
| 3 | A03 | DPL0733 (72841366-72841554) |
| 3 | A03 | DPL0170 (74227146-74227379) |
| 3 | A03 | BNL2689 (74242106-74242218) |
| 3 | A03 | TMB0253 (75236410-75236590) |
| 3 | A03 | MUCS582 (82049324-82049602) |
| 3 | A03 | NAU3239 (86325788-86325985) |
| 3 | A03 | NAU2336 (89572696-89572867) |
| 3 | A03 | NAU0998 (93482494-93482659) |
| 3 | A03 | CIR0263 (94856421-94856513) |
| 3 | A03 | NAU4022 (94944856-94945033) |
| 3 | A03 | HAU1322 (97186559-97186799) |
| 3 | A03 | BNL3259 (97427110-97427319) |
| 3 | A03 | NAU2161 (99227817-99228085) |
| 3 | A03 | MUSB0969 OR BNL3267 OR NAU3808 |
| 4 | A04 | NAU3592 (1737745-1737906) |
| 4 | A04 | CIR344 (1781240-1781357) |
| 4 | A04 | DPL0573 (4113280-4113448) |
| 4 | A04 | HAU1785 (4443453-4443683) |
| 4 | A04 | BNL3496 (4804742-4804917) |
| 4 | A04 | DPL0299 (9040817-9041003) |
| 4 | A04 | BNL4047 (10053242-10053399) |
| 4 | A04 | NAU3205 (11684816-11685027) |
| 4 | A04 | BNL3807 (14878788-14879000) |
| 4 | A04 | MUSB1015 (14974479-14974760) |
| 4 | A04 | TMB0728 (17907895-17908110) |
| 4 | A04 | DPL0273 (27329052-27329270) |
| 4 | A04 | NAU5180 (29802896-29803097) |
| 4 | A04 | TMB0872 (31388114-31388318) |
| 4 | A04 | MUSS0027 (47724897-47725175) |
| 4 | A04 | NAU2782 (48817689-48817934) |
| 4 | A04 | MUSS0187 (52443654-52443916) |
| 4 | A04 | DPL0085 (55915257-55915483) |
| 4 | A04 | DPL0667 (56709074-56709285) |
| 4 | A04 | NAU3093 (59166040-59166290) |
| 4 | A04 | CIR0122 (60683992-60684131) |
| 5 | A05 | NAU1068 (253507-253750) |
| 5 | A05 | NAU3245 (617148-617365) |
| 5 | A05 | TMB1418 (1289672-1289863) |
| 5 | A05 | MUSB0799 (2049078-2049364) |
| 5 | A05 | HAU2846 (2053513-2053651) |
| 5 | A05 | TMB1296 (2369694-2369927) |
| 5 | A05 | DPL0641 (2369806-2369947) |
| 5 | A05 | NAU2894 (4627546-4627708) |
| 5 | A05 | BNL4071 (8097803-8098033) |
| 5 | A05 | DPL0241 (9708308-9708434) |
| 5 | A05 | DPL0177 (11564380-11564623) |
| 5 | A05 | DPL0368 (11664667-11664914) |
| 5 | A05 | BNL3569 (13503638-13503792) |
| 5 | A05 | BNL3903 (16664114-16664265) |
| 5 | A05 | NAU5486 (17378639-17378879) |
| 5 | A05 | NAU2029 (18760055-18760239) |
| 5 | A05 | NAU3001 (22777101-22777323) |
| 5 | A05 | NAU3620 (22777192-22777449) |
| 5 | A05 | CIR0240 (23535284-23535526) |
| 5 | A05 | BNL2656 (24967164-24967332) |
| 5 | A05 | NAU4042 (28825759-28825928) |
| 5 | A05 | JESPR0241 (29801688-29801885) |
| 5 | A05 | NAU4896 (32102388-32102539) |
| 5 | A05 | BNL2749 (36209457-36209609) |
| 5 | A05 | MUSB1043 (38258967-38259169) |
| 5 | A05 | JESPR0042 (39686686-39686827) |
| 5 | A05 | MUSB1034 (41166269-41166519) |
| 5 | A05 | HAU0006 (43592402-43592602) |
| 5 | A05 | MUSB0059 (46503600-46503830) |
| 5 | A05 | MUSB0904 (49633005-49633422) |
| 5 | A05 | TMB1888 (50370631-50370965) |
| 5 | A05 | CIR0301 (54090990-54091119) |
| 5 | A05 | NAU4040 (56755341-56755517) |
| 5 | A05 | DPL0163 (57090014-57090178) |
| 5 | A05 | NAU3139 (64552600-64552773) |
| 5 | A05 | NAU2296 (65523147-65523261) |
| 5 | A05 | NAU5099 (70499986-70500204) |
| 5 | A05 | BNL3995 (73016021-73016218) |
| 5 | A05 | DPL0637 (73620697-73620822) |
| 5 | A05 | BNL3241 (76574675-76574798) |
| 5 | A05 | JESPR0065 (80780184-80780326) |
| 5 | A05 | CIR0185 (84369963-84370091) |
| 5 | A05 | BNL2732 (84793327-84793537) |
| 5 | A05 | NAU3557 (86152009-86152264) |
| 5 | A05 | BNL0448 (86777392-86777597) |
| 5 | A05 | MUSS0219 (91380795-91381022) |
| 6 | A06 | CIR0203 (2321499-2321756) |
| 6 | A06 | NAU5270 (4151149-4151331) |
| 6 | A06 | DPL0702 (4772010-4772211) |
| 6 | A06 | CIR0280 (5184279-5184502) |
| 6 | A06 | HAU1355 (6545924-6546186) |
| 6 | A06 | HAU3157 (8306892-8307189) |
| 6 | A06 | HAU0615 (10679767-10680055) |
| 6 | A06 | DPL0847 (10682879-10683065) |
| 6 | A06 | BNL3250 (19217067-19217228) |
| 6 | A06 | DPL0874 (19721516-19721707) |
| 6 | A06 | DPL0507 (21866897-21867337) |
| 6 | A06 | BNL1440 (27967417-27967676) |
| 6 | A06 | DPL0239 (30632209-30632450) |
| 6 | A06 | BNL1153 (30783707-30784024) |
| 6 | A06 | TMB1484 (35892085-35892324) |
| 6 | A06 | TMB1725 (36582661-36582837) |
| 6 | A06 | MUSB0847 (38931722-38931917) |
| 6 | A06 | MUSB0550 (40027027-40027337) |
| 6 | A06 | MUSB0754 (41651369-41651664) |
| 6 | A06 | BNL3937 (43846879-43847110) |
| 6 | A06 | TMB2958 (44033466-44033706) |
| 6 | A06 | MUSB0971 (57216288-57216636) |
| 6 | A06 | MUSB0634 (65397784-65398114) |
| 6 | A06 | MUSB0799 (65397797-65398084) |
| 6 | A06 | BNL3103 (68265534-68265737) |
| 6 | A06 | CIR0150 (69136538-69136664) |
| 6 | A06 | HAU1931 (75728512-75728766) |
| 6 | A06 | DPL0566 (80865022-80865221) |
| 6 | A06 | MUSB0309 (81421482-81421637) |
| 6 | A06 | NAU5433 (89924614-89924834) |
| 6 | A06 | NAU5373 (90713304-90713493) |
| 6 | A06 | DPL0811 (95754540-95754756) |
| 6 | A06 | BNL2691 (97732574-97732792) |
| 6 | A06 | BNL2884 (98075649-98075815) |
| 6 | A06 | DPL0282 (100622933-100623167) |
| 6 | A06 | DPL0617 (101677474-101677653) |
| 7 | A07 | NAU5152 (4015733-4015954) |
| 7 | A07 | NAU2931 (7037245-7037421) |
| 7 | A07 | CIR0412 (7745259-7745426) |
| 7 | A07 | NAU1048 (7869173-7869395) |
| 7 | A07 | NAU4956 (8071564-8071826) |
| 7 | A07 | BNL2634 (9292446-9292641) |
| 7 | A07 | NAU5408 (13196183-13196338) |
| 7 | A07 | NAU2620 (13768717-13768956) |
| 7 | A07 | NAU2627 (14563594-14563805) |
| 7 | A07 | MUSB1181 (15407296-15407468) |
| 7 | A07 | MUSS0006 (20824626-20824911) |
| 7 | A07 | NAU5061 (26942547-26942824) |
| 7 | A07 | HAU1780 (28746896-28747109) |
| 7 | A07 | BNL3415 (29801268-29801505) |
| 7 | A07 | CIR0141 (31229099-31229246) |
| 7 | A07 | NAU2995 (32291019-32291194) |
| 7 | A07 | NAU5024 (35008226-35008495) |
| 7 | A07 | DPL0294 (35894179-35894337) |
| 7 | A07 | DPL0287 (36336834-36337015) |
| 7 | A07 | BNL1694 (38823109-38823348) |
| 7 | A07 | DPL0223 (39731923-39732120) |
| 7 | A07 | BNL1122 (42904368-42904537) |
| 7 | A07 | DPL0013 (42904374-42904549) |
| 7 | A07 | DPL0061 (44546820-44547036) |
| 7 | A07 | NAU1305 (45549916-45550127) |
| 7 | A07 | CIR0245 (49056693-49056892) |
| 7 | A07 | MUSB0971 (49204755-49205095) |
| 7 | A07 | DPL0048 (51023825-51024048) |
| 7 | A07 | TMB0180 (51903558-51903757) |
| 7 | A07 | MUSB1043 (54582020-54582225) |
| 7 | A07 | TMB2566 (55275429-55275613) |
| 7 | A07 | HAU1510 (57515139-57515265) |
| 7 | A07 | DPL0652 (64885462-64885707) |
| 7 | A07 | TMB2944 (67083063-67083302) |
| 7 | A07 | TMB0004 (67732882-67733085) |
| 7 | A07 | HAU2662 (73015986-73016290) |
| 7 | A07 | HAU1399 (73340951-73341129) |
| 7 | A07 | NAU966 (77560572-77560762) |
| 8 | A08 | MUSS0409 (1155486-1155700) |
| 8 | A08 | MUCS148 (1654500-1654785) |
| 8 | A08 | NAU1209 (2883564-2883737) |
| 8 | A08 | MUSS0021 (3714904-3715029) |
| 8 | A08 | NAU1350 (5750744-5750977) |
| 8 | A08 | BNL0252 (7117568-7117730) |
| 8 | A08 | NAU2829 (9857572-9857712) |
| 8 | A08 | MUSB0442 (14556908-14557254) |
| 8 | A08 | MUSB0087 (16384215-16384459) |
| 8 | A08 | DPL0755 (17010218-17010409) |
| 8 | A08 | MUSB1018 (20726460-20726662) |
| 8 | A08 | DPL0839 (21087871-21088053) |
| 8 | A08 | MUSB0799 (23937796-23938083) |
| 8 | A08 | MUSB0749 (24140812-24141109) |
| 8 | A08 | MUSB1018 (26211006-26211208) |
| 8 | A08 | DPL0146 (30610853-30611039) |
| 8 | A08 | NAU3632 (35409927-35410128) |
| 8 | A08 | HAU2522 (53021839-53021995) |
| 8 | A08 | BNL1017 (65640935-65641076) |
| 8 | A08 | NAU3207 (67505774-67506017) |
| 8 | A08 | NAU5173 (81408364-81408633) |
| 8 | A08 | CIR0082 (81828276-81828445) |
| 8 | A08 | NAU2434 (84240512-84240749) |
| 8 | A08 | NAU0891 (84240559-84240758) |
| 8 | A08 | DPL0398 (84358531-84358772) |
| 8 | A08 | MUSB0799 (84808296-84808583) |
| 8 | A08 | DPL0176 (89603456-89603690) |
| 8 | A08 | NAU3424 (90401866-90402070) |
| 8 | A08 | NAU3964 (90808962-90809138) |
| 8 | A08 | BNL2961 (90946008-90946244) |
| 8 | A08 | NAU3201 (94091584-94091817) |
| 8 | A08 | BNL3556 (96538060-96538198) |
| 8 | A08 | CIR0237 (96786067-96786195) |
| 8 | A08 | DPL0068 (96813234-96813400) |
| 8 | A08 | HAU1567 (102099020-102099330) |
| 8 | A08 | CIR0244 (103026054-103026180) |
| 9 | A09 | DPL0222 (1400513-1400753) |
| 9 | A09 | NAU3888 (2448616-2448864) |
| 9 | A09 | HAU2496 (2448653-2448920) |
| 9 | A09 | MUSS0506 (3104397-3104620) |
| 9 | A09 | TMB2279 (4290009-4290250) |
| 9 | A09 | BNL1707 (5916463-5916618) |
| 9 | A09 | DPL0679 (8340382-8340559) |
| 9 | A09 | DPL0618 (9388356-9388545) |
| 9 | A09 | DPL0854 (10932787-10933028) |
| 9 | A09 | NAU2575 (13486585-13486767) |
| 9 | A09 | NAU3732 (13503016-13503214) |
| 9 | A09 | TMB0109 (21543056-21543260) |
| 9 | A09 | CIR0353 (21543093-21543257) |
| 9 | A09 | DPL0546 (22652427-22653632) |
| 9 | A09 | MUSB1034 (25968900-25969153) |
| 9 | A09 | MUSB0995 (26364806-26365080) |
| 9 | A09 | MUSB0907 (31464489-31464690) |
| 9 | A09 | DPL0550 (32488859-32489073) |
| 9 | A09 | MUSB0087 (41049207-41049453) |
| 9 | A09 | NAU3194 (42873842-42874018) |
| 9 | A09 | NAU2832 (48275241-48275827) |
| 9 | A09 | BNL3779 (53582633-53582852) |
| 9 | A09 | BNL3511 (54664611-54664775) |
| 9 | A09 | BNL1672 (55137528-55137633) |
| 9 | A09 | BNL3410 (59918229-59918446) |
| 9 | A09 | MUSS0579 (60459984-60460157) |
| 9 | A09 | NAU2709 (60718662-60718857) |
| 9 | A09 | NAU3966 (61548314-61548687) |
| 9 | A09 | MUSS0266 (63151250-63151390) |
| 9 | A09 | NAU3738 (67994162-67994386) |
| 9 | A09 | NAU0858 (68307219-68307417) |
| 9 | A09 | BNL1414 (68726991-68727129) |
| 9 | A09 | DPL0395 (72275569-72275780) |
| 10 | A10 | NAU2776 (1862589-1862822) |
| 10 | A10 | HAU0894 (4921499-4921698) |
| 10 | A10 | DPL0149 (7705183-7705333) |
| 10 | A10 | BNL0256 (7782816-7783023) |
| 10 | A10 | MUSB0808 (8961642-8961867) |
| 10 | A10 | BNL3300 (11894853-11894972) |
| 10 | A10 | TMB1630 (12047880-12048110) |
| 10 | A10 | HAU0635 (12126120-12126312) |
| 10 | A10 | BNL3499 (13456900-13457117) |
| 10 | A10 | BNL1160 (15959036-15959408) |
| 10 | A10 | BNL2631 (16607438-16607629) |
| 10 | A10 | NAU904 (19096715-19096900) |
| 10 | A10 | HAU1969 (20563207-20563439) |
| 10 | A10 | MUSB0799 (22425480-22425768) |
| 10 | A10 | MUSB0319 (22459257-22459561) |
| 10 | A10 | MUSB0799 (25084388-25084671) |
| 10 | A10 | MUSB1018 (25268267-25268469) |
| 10 | A10 | HAU3201 (26629023-26629246) |
| 10 | A10 | NAU3013 (26661547-26661691) |
| 10 | A10 | CIR0400 (30483337-30483490) |
| 10 | A10 | MUSB0749 (34245560-34245857) |
| 10 | A10 | MUSB1018 (35916698-35916900) |
| 10 | A10 | MUSB0995 (36109321-36109599) |
| 10 | A10 | BNL3670 (38211883-38212020) |
| 10 | A10 | MUSB0749 (41946221-41946517) |
| 10 | A10 | HAU2178 (42053743-42053975) |
| 10 | A10 | MUSS414 (42053831-42054130) |
| 10 | A10 | HAU3156 (46994987-46995231) |
| 10 | A10 | MUCS283 (49321188-49321343) |
| 10 | A10 | MUCS283 (49336287-49336442) |
| 10 | A10 | DPL0431 (60612984-60613186) |
| 10 | A10 | DPL0507 (62494219-62494381) |
| 10 | A10 | BNL3895 (62739012-62739185) |
| 10 | A10 | MUSB0930 (65525702-65525922) |
| 10 | A10 | MUSB0847 (68727349-68727543) |
| 10 | A10 | MUSB1168 (73676207-73676539) |
| 10 | A10 | NAU1041 (76517970-76518209) |
| 10 | A10 | NAU5438 (81432519-81432765) |
| 10 | A10 | BNL3790 (86689893-86690054) |
| 10 | A10 | BNL3563 (89108333-89108561) |
| 10 | A10 | HAU0949 (89153813-89154102) |
| 10 | A10 | BNL2960 (91231768-91231917) |
| 10 | A10 | NAU1280 (94060744-94060946) |
| 10 | A10 | NAU2991 (94513498-94513716) |
| 10 | A10 | NAU5323 (97861406-97861625) |
| 10 | A10 | NAU4921 (98385800-98386168) |
| 11 | A11 | NAU5461 (1445218-1445424) |
| 11 | A11 | NAU5192 (2035349-2035568) |
| 11 | A11 | MUSB1197 (3998366-3998714) |
| 11 | A11 | NAU2118 (5665913-5666150) |
| 11 | A11 | NAU967 (5695375-5695581) |
| 11 | A11 | NAU5354 (5695419-5695662) |
| 11 | A11 | DPL0585 (8598826-8598961) |
| 11 | A11 | BNL3449 (8944415-8944555) |
| 11 | A11 | BNL1151 (11613896-11614070) |
| 11 | A11 | CIR0399 (12969240-12969348) |
| 11 | A11 | BNL1404 (13461772-13462001) |
| 11 | A11 | JESPR0135 (13461869-13462001) |
| 11 | A11 | BNL2812 (15431404-15431544) |
| 11 | A11 | MUSB0550 (16209298-16209610) |
| 11 | A11 | HAU3344 (16949678-16949968) |
| 11 | A11 | HAU2004 (16995376-16995650) |
| 11 | A11 | DPL0199 (24633299-24633548) |
| 11 | A11 | NAU5212 (26897966-26898224) |
| 11 | A11 | NAU2950 (27357541-27357698) |
| 11 | A11 | BNL2632 (33647478-33647687) |
| 11 | A11 | HAU1295 (34189371-34189601) |
| 11 | A11 | TMB0359 (46758934-46759109) |
| 11 | A11 | BNL2805 (49637579-49637812) |
| 11 | A11 | TMB2453 (50356790-50356976) |
| 11 | A11 | MUSS0092 (54537588-54537862) |
| 11 | A11 | BNL3282 (55685287-55685449) |
| 11 | A11 | HAU1756 (56902955-56903088) |
| 11 | A11 | TMB0242 (58846664-58846893) |
| 11 | A11 | CIR0068 (58871933-58872098) |
| 11 | A11 | DPL0215 (62333639-62333842) |
| 11 | A11 | MUSB1018 (63660818-63661020) |
| 11 | A11 | BNL2906 (63926127-63926301) |
| 11 | A11 | NAU2877 (70130434-70130598) |
| 11 | A11 | DPL0338 (71968816-71969012) |
| 11 | A11 | TMB0728 (76318675-76318893) |
| 11 | A11 | DPL0325 (77081202-77081355) |
| 11 | A11 | NAU3704 (79024961-79025166) |
| 11 | A11 | NAU4086 (80368894-80369195) |
| 11 | A11 | NAU3158 (82713382-82713607) |
| 11 | A11 | HAU3394 (82713518-82713687) |
| 11 | A11 | NAU3493 (82740107-82740277) |
| 11 | A11 | NAU5505 (83746008-83746206) |
| 11 | A11 | DPL0209 (85199008-85199210) |
| 11 | A11 | NAU2152 (88794811-88795008) |
| 11 | A11 | DPL0475 (91100077-91100273) |
| 12 | A12 | NAU3561 (2105174-2105422) |
| 12 | A12 | CIR0085 (3821796-3822016) |
| 12 | A12 | CIR085 (3821796-3822016) |
| 12 | A12 | BNL4041 (4243085-4243298) |
| 12 | A12 | NAU3897 (5050187-5050347) |
| 12 | A12 | BNL3261 (6604698-6604898) |
| 12 | A12 | NAU0877 (7910851-7911050) |
| 12 | A12 | NAU877 (7910851-7911050) |
| 12 | A12 | CIR167 (8116705-8116908) |
| 12 | A12 | NAU3666 (9655233-9655437) |
| 12 | A12 | DPL0039 (10729166-10729401) |
| 12 | A12 | NAU2902 (14323824-14324034) |
| 12 | A12 | BNL3867 (16438318-16438439) |
| 12 | A12 | NAU3442 (18025689-18025847) |
| 12 | A12 | BNL0116 (22815792-22815932) |
| 12 | A12 | BNL3250 (24456618-24456766) |
| 12 | A12 | BNL0840 (26579937-26580136) |
| 12 | A12 | TMB0573 (26579938-26580136) |
| 12 | A12 | MUSB0799 (34260480-34260767) |
| 12 | A12 | MUSB0907 (34990943-34991147) |
| 12 | A12 | MUSB1043 (37346001-37346206) |
| 12 | A12 | MUSB0846 (38738525-38738736) |
| 12 | A12 | BNL1041 (39060387-39060541) |
| 12 | A12 | NAU3812 (41813608-41813807) |
| 12 | A12 | MUSS0018 (45747968-45748293) |
| 12 | A12 | MUSB0799 (52280156-52280443) |
| 12 | A12 | MUSB1307 (56624547-56624888) |
| 12 | A12 | DPL0280 (61826660-61826876) |
| 12 | A12 | NAU1278 (61961237-61961467) |
| 12 | A12 | NAU3961 (63923916-63924121) |
| 12 | A12 | BNL0391 (66072939-66073245) |
| 12 | A12 | BNL1673 (70041737-70041933) |
| 12 | A12 | NAU5419 (71979010-71979237) |
| 12 | A12 | NAU0943 (72319754-72319934) |
| 12 | A12 | NAU943 (72319754-72319934) |
| 12 | A12 | NAU3293 (74399628-74399771) |
| 12 | A12 | NAU3294 (74399746-74399907) |
| 12 | A12 | BNL1679 (76438142-76438305) |
| 12 | A12 | NAU2715 (76512966-76513155) |
| 12 | A12 | NAU2696 (77768423-77768616) |
| 12 | A12 | NAU5047 (80686021-80686270) |
| 12 | A12 | NAU5204 (83257388-83257654) |
| 12 | A12 | NAU3778 (85473461-85473682) |
| 13 | A13 | NAU3189 (4015906-4016096) |
| 13 | A13 | DPL0249 (5632113-5632232) |
| 13 | A13 | MUSB0812 (5704700-5705064) |
| 13 | A13 | HAU2558 (12739596-12739728) |
| 13 | A13 | CIR0096 (14036435-14036763) |
| 13 | A13 | MUSS0010 (14409362-14409624) |
| 13 | A13 | TMB2108 (23601860-23602017) |
| 13 | A13 | DPL0223 (27574762-27574980) |
| 13 | A13 | BNL4079 (28753687-28753841) |
| 13 | A13 | MUSB0309 (30102883-30103037) |
| 13 | A13 | NAU2381 (33476731-33476894) |
| 13 | A13 | MUSB0972 (34552731-34552913) |
| 13 | A13 | MUSB0564 (35172216-35172655) |
| 13 | A13 | MUSB0995 (35172341-35172612) |
| 13 | A13 | NAU3989 (37876527-37876774) |
| 13 | A13 | NAU1023 (38440510-38440711) |
| 13 | A13 | NAU3570 (39100814-39101037) |
| 13 | A13 | HAU1091 (39180284-39180383) |
| 13 | A13 | MUSB0930 (41285995-41286220) |
| 13 | A13 | TMB1369 (44032929-44033114) |
| 13 | A13 | TMB1603 (45782955-45783166) |
| 13 | A13 | BNL2762 (47248580-47248828) |
| 13 | A13 | HAU3061 (47687726-47687934) |
| 13 | A13 | BNL1555 (57524995-57525227) |
| 13 | A13 | BNL4029 (58652724-58652970) |
| 13 | A13 | NAU5364 (68081952-68082167) |
| 13 | A13 | NAU3017 (69863804-69864042) |
| 13 | A13 | MUSS0140 (70850732-70851013) |
| 13 | A13 | MUCS0145 (73365335-73365506) |
| 13 | A13 | MUCS145 (73365335-73365506) |
| 13 | A13 | MUCS0267 (74370511-74370806) |
| 13 | A13 | MUCS267 (74370511-74370806) |
| 13 | A13 | NAU4871 (75819056-75819199) |
| 13 | A13 | NAU3080 (76802091-76802318) |
| 13 | A13 | NAU2038 (77671681-77672209) |
| 13 | A13 | NAU3843 (77786545-77786732) |
| 14 | D02 | TMB0071 (1461099-1461259) |
| 14 | D02 | BNL3644 (1485298-1485486) |
| 14 | D02 | JESPR0293 (7505796-7505892) |
| 14 | D02 | NAU4024 (10085323-10085516) |
| 14 | D02 | MUCS494 (12354819-12355085) |
| 14 | D02 | TMB1513 (12625299-12625547) |
| 14 | D02 | TMB2953 (14958381-14958618) |
| 14 | D02 | NAU3058 (22601711-22602215) |
| 14 | D02 | BNL3523 (23513409-23513601) |
| 14 | D02 | NAU3439 (27605553-27605776) |
| 14 | D02 | BNL3267 (29663912-29664069) |
| 14 | D02 | DPL0242 (34638424-34638621) |
| 14 | D02 | DPL0734 (40282978-40283193) |
| 14 | D02 | NAU3120 (45036475-45036688) |
| 14 | D02 | TMB0324 (45268957-45269161) |
| 14 | D02 | TMB0607 (47073426-47073765) |
| 14 | D02 | HAU1527 (48371179-48371354) |
| 14 | D02 | HAU0438 (52101458-52101738) |
| 14 | D02 | DPL0565 (55661766-55662118) |
| 14 | D02 | CIR0239 (57258643-57258804) |
| 14 | D02 | NAU2272 (59025671-59025836) |
| 14 | D02 | BNL3034 (59793741-59793897) |
| 15 | D01 | NAU4073 (54148-54313) |
| 15 | D01 | HAU0282 (134771-134872) |
| 15 | D01 | HAU1721 (317624-317865) |
| 15 | D01 | CIR0110 (378268-378390) |
| 15 | D01 | BNL0846 (398631-398845) |
| 15 | D01 | HAU1045 (521112-521411) |
| 15 | D01 | HAU2307 (521254-521558) |
| 15 | D01 | NAU3102 (4813428-4813634) |
| 15 | D01 | DPL0051 (5403845-5404070) |
| 15 | D01 | NAU3057 (6650815-6651002) |
| 15 | D01 | NAU5172 (10455752-10455935) |
| 15 | D01 | BNL3090 (11423679-11423893) |
| 15 | D01 | BNL4095 (13239347-13239562) |
| 15 | D01 | BNL1350 (16909514-16909725) |
| 15 | D01 | CIR0085 (21385338-21385552) |
| 15 | D01 | BNL2564 (22968758-22968880) |
| 15 | D01 | BNL2700 (26295322-26295479) |
| 15 | D01 | BNL3902 (26803236-26803427) |
| 15 | D01 | NAU3384 (27772144-27772335) |
| 15 | D01 | TMB0375 (28143551-28143765) |
| 15 | D01 | MUSB1121 (29485733-29486062) |
| 15 | D01 | NAU2985 (32920292-32920513) |
| 15 | D01 | DPL0615 (35572280-35572515) |
| 15 | D01 | BNL2646 (38576039-38576181) |
| 15 | D01 | BNL4082 (39921024-39921196) |
| 15 | D01 | MUSB1079 (40380398-40380530) |
| 15 | D01 | BNL3652 (45206800-45206952) |
| 15 | D01 | JESPR0205 (46125254-46125360) |
| 15 | D01 | NAU3690 (49694283-49694452) |
| 15 | D01 | BNL2700 (50956024-50956176) |
| 15 | D01 | NAU3433 (53813347-53813830) |
| 15 | D01 | MUCS0164 (61251005-61251608) |
| 15 | D01 | MUCS164 (61251005-61251608) |
| 16 | D07 | NAU5325 (65946-66151) |
| 16 | D07 | NAU3826 (2624947-2625366) |
| 16 | D07 | NAU3053 (3460341-3460522) |
| 16 | D07 | BNL3452 (3777082-3777272) |
| 16 | D07 | NAU5120 (6295751-6295915) |
| 16 | D07 | MUCS0616 (7135286-7135498) |
| 16 | D07 | NAU3380 (11290803-11291170) |
| 16 | D07 | JESPR0102 (12136166-12136274) |
| 16 | D07 | BNL3008 (12556324-12556456) |
| 16 | D07 | TMB1409 (14119438-14119632) |
| 16 | D07 | NAU5061 (20842673-20842959) |
| 16 | D07 | HAU1780 (21971880-21972093) |
| 16 | D07 | CIR0141 (23598220-23598367) |
| 16 | D07 | JESPR0228 (24296077-24296325) |
| 16 | D07 | DPL0223 (27574762-27574980) |
| 16 | D07 | BNL1122 (29324861-29325024) |
| 16 | D07 | TMB2566 (36666611-36666771) |
| 16 | D07 | HAU1510 (38350055-38350187) |
| 16 | D07 | HAU1205 (38976789-38977000) |
| 16 | D07 | HAU0033 (38976827-38977004) |
| 16 | D07 | DPL0897 (40917280-40917451) |
| 16 | D07 | HAU2113 (41640054-41640304) |
| 16 | D07 | DPL0385 (42570667-42570895) |
| 16 | D07 | JESPR0297 (42647166-42647314) |
| 16 | D07 | NAU3180 (43621343-43621567) |
| 17 | D03 | CIR0251 (4764482-4764667) |
| 17 | D03 | DPL0073 (6189956-6190178) |
| 17 | D03 | NAU3626 (10102632-10102868) |
| 17 | D03 | NAU3875 (13270450-13270642) |
| 17 | D03 | BNL3590 (26940735-26940924) |
| 17 | D03 | BNL4003 (27873611-27873726) |
| 17 | D03 | NAU3639 (29218495-29218664) |
| 17 | D03 | MUSB0964 (30862078-30862419) |
| 17 | D03 | DPL0279 (32840621-32840770) |
| 17 | D03 | NAU3808 (32856752-32856957) |
| 17 | D03 | BNL4073 (34552108-34552224) |
| 17 | D03 | BNL3371 (36864150-36864344) |
| 17 | D03 | DPL0529 (38033222-38033394) |
| 17 | D03 | BNL1606 (41488231-41488423) |
| 17 | D03 | DPL0045 (41488304-41488523) |
| 17 | D03 | BNL2496B (43885635-43885785) |
| 17 | D03 | NAU2031 (44025664-44025897) |
| 17 | D03 | HAU0292 (44792112-44792353) |
| 17 | D03 | NAU2691 (45752792-45753036) |
| 17 | D03 | NAU2836 (46154907-46155109) |
| 18 | D13 | HAU1577 (457674-457870) |
| 18 | D13 | TMB2762 (1428317-1428531) |
| 18 | D13 | BNL2544 (3272111-3272328) |
| 18 | D13 | DPL0249 (5361908-5362036) |
| 18 | D13 | BNL3558 (5627320-5627529) |
| 18 | D13 | CIR0096 (7049600-7049924) |
| 18 | D13 | HAU0083 (7367201-7367349) |
| 18 | D13 | NAU4105 (8707661-8707857) |
| 18 | D13 | BNL2652 (11827341-11827529) |
| 18 | D13 | DPL0033 (12172612-12172739) |
| 18 | D13 | BNL4035 (12530076-12530289) |
| 18 | D13 | BNL4079 (16712116-16712266) |
| 18 | D13 | TMB0114 (24593300-24593444) |
| 18 | D13 | NAU3534 (27110995-27111210) |
| 18 | D13 | BNL1079 (28947936-28948095) |
| 18 | D13 | BNL1721 (31695923-31696109) |
| 18 | D13 | NAU5273 (33691219-33691453) |
| 18 | D13 | NAU3427 (36293244-36293428) |
| 18 | D13 | BNL4029 (40354132-40354524) |
| 18 | D13 | NAU5392 (42604122-42604356) |
| 18 | D13 | NAU2443 (45316005-45316134) |
| 18 | D13 | NAU5109 (50372936-50373381) |
| 18 | D13 | MUSS0603 (51883132-51883416) |
| 18 | D13 | MUCS267 (54178220-54178506) |
| 18 | D13 | NAU3321 (57071756-57072003) |
| 18 | D13 | MUSB1135 (59192409-59192557) |
| 18 | D13 | DPL0922 (59316884-59317104) |
| 19 | D05 | NAU3405 (2240880-2241064) |
| 19 | D05 | DPL0140 (3366540-3366784) |
| 19 | D05 | NAU5387 (4782856-4783114) |
| 19 | D05 | CIR0212 (7735484-7735640) |
| 19 | D05 | BNL1690 (8961842-8961957) |
| 19 | D05 | HAU1969 (10493298-10493538) |
| 19 | D05 | BNL0285 (10511369-10511613) |
| 19 | D05 | NAU1221 (13668910-13669151) |
| 19 | D05 | NAU2126 (14447694-14447879) |
| 19 | D05 | NAU5160 (15387287-15387417) |
| 19 | D05 | BNL3903 (16212058-16212219) |
| 19 | D05 | NAU3325 (17020722-17020935) |
| 19 | D05 | DPL0156 (17346076-17346305) |
| 19 | D05 | DPL0495 (19136715-19136928) |
| 19 | D05 | NAU2741 (20922577-20922840) |
| 19 | D05 | BNL3948 (21363805-21363905) |
| 19 | D05 | BNL0390 (21843356-21843591) |
| 19 | D05 | BNL3992 (23300201-23300331) |
| 19 | D05 | BNL3977 (23858393-23858522) |
| 19 | D05 | NAU5489 (26313345-26313586) |
| 19 | D05 | NAU2274 (27929549-27929652) |
| 19 | D05 | NAU4896 (29369465-29369616) |
| 19 | D05 | NAU2014 (30625545-30625776) |
| 19 | D05 | JESPR0001 (32235984-32236434) |
| 19 | D05 | NAU3205 (50573695-50573906) |
| 19 | D05 | BNL1671 (52346543-52346670) |
| 19 | D05 | CIR0222 (54287946-54288250) |
| 19 | D05 | BNL3347 (55139326-55139486) |
| 19 | D05 | BNL2821 (56652933-56653126) |
| 19 | D05 | DPL0573 (57576419-57576593) |
| 19 | D05 | NAU2801 (58565388-58565609) |
| 20 | D10 | NAU2776 (1645800-1646033) |
| 20 | D10 | HAU2021 (2921671-2921888) |
| 20 | D10 | DPL0297 (3018985-3019204) |
| 20 | D10 | NAU5359 (4129879-4130223) |
| 20 | D10 | NAU0904 (11073380-11073562) |
| 20 | D10 | NAU4973 (12446148-12446355) |
| 20 | D10 | BNL2631 (12775954-12776137) |
| 20 | D10 | MUSB1168 (16826465-16826786) |
| 20 | D10 | NAU1005 (19607383-19607583) |
| 20 | D10 | BNL3948 (21363805-21363905) |
| 20 | D10 | BNL3379 (22979090-22979286) |
| 20 | D10 | TMB1687 (27123150-27123353) |
| 20 | D10 | BNL3660 (28985349-28985522) |
| 20 | D10 | TMB0281 (35892674-35892830) |
| 20 | D10 | BNL3993 (39957789-39958000) |
| 20 | D10 | BNL2689 (40913405-40913513) |
| 20 | D10 | TMB0987 (46527145-46527341) |
| 20 | D10 | NAU4928 (49099297-49099513) |
| 20 | D10 | BNL3071 (50642526-50642688) |
| 20 | D10 | NAU4881 (50709477-50709728) |
| 20 | D10 | DPL0225 (51261580-51261821) |
| 20 | D10 | NAU2869 (51533669-51533906) |
| 20 | D10 | BNL0169 (54834494-54834692) |
| 21 | D11 | HAU0618 (3240240-3240532) |
| 21 | D11 | NAU3074 (5890500-5890680) |
| 21 | D11 | BNL1034 (6193803-6194022) |
| 21 | D11 | NAU2998 (6892390-6892574) |
| 21 | D11 | DPL0062 (7280851-7280984) |
| 21 | D11 | NAU3381 (8219709-8219911) |
| 21 | D11 | DPL0585 (8703144-8703261) |
| 21 | D11 | TMB1642 (8792984-8793222) |
| 21 | D11 | BNL3418 (10391071-10391215) |
| 21 | D11 | BNL1151 (10978009-10978167) |
| 21 | D11 | DPL0181 (11081484-11081632) |
| 21 | D11 | CIR0385 (11563297-11563419) |
| 21 | D11 | BNL1404 (12317135-12317349) |
| 21 | D11 | MUSB0155 (14225132-14225254) |
| 21 | D11 | NAU5418 (15684034-15684282) |
| 21 | D11 | DPL0199 (20414286-20414550) |
| 21 | D11 | NAU2950 (22158809-22158965) |
| 21 | D11 | BNL2632 (24916187-24916391) |
| 21 | D11 | BNL3997 (25572930-25573104) |
| 21 | D11 | NAU2758 (28393184-28393352) |
| 21 | D11 | TMB0426 (40296088-40296286) |
| 21 | D11 | NAU1014 (43005589-43005756) |
| 21 | D11 | BNL0261 (47701989-47702214) |
| 21 | D11 | BNL1408 (49093445-49093658) |
| 21 | D11 | DPL0338 (50217587-50217768) |
| 21 | D11 | BNL1551 (54771881-54772064) |
| 21 | D11 | TMB1276 (54995591-54995838) |
| 21 | D11 | NAU4086 (55250801-55250988) |
| 21 | D11 | NAU3158 (57209940-57210165) |
| 21 | D11 | BNL3279 (59769332-59769457) |
| 21 | D11 | NAU2152 (61712786-61713008) |
| 21 | D11 | NAU5428 (62389615-62389830) |
| 21 | D11 | NAU3740 (63140073-63140220) |
| 21 | D11 | BNL2906 (63695716-63695882) |
| 21 | D11 | DPL0475 (63951709-63951892) |
| 22 | D04 | MUSS0219 (706914-707141) |
| 22 | D04 | DPL0722 (2872102-2872256) |
| 22 | D04 | BNL0448 (4296403-4296618) |
| 22 | D04 | BNL4092 (5596915-5597123) |
| 22 | D04 | DPL0055 (7725760-7725866) |
| 22 | D04 | BNL3601 (10562323-10562497) |
| 22 | D04 | NAU3824 (13730160-13730397) |
| 22 | D04 | BNL4015 (15248773-15248890) |
| 22 | D04 | DPL0810 (15707998-15708157) |
| 22 | D04 | NAU3633 (21617585-21617788) |
| 22 | D04 | HAU1332 (22555512-22555744) |
| 22 | D04 | NAU3491 (23489346-23489560) |
| 22 | D04 | MUSB1093 (29365514-29365798) |
| 22 | D04 | BNL3994 (34332684-34332953) |
| 22 | D04 | DPL0489 (37322879-37323074) |
| 22 | D04 | MUSB1050 (39865327-39865524) |
| 22 | D04 | DPL0085 (44241365-44241593) |
| 22 | D04 | BNL0358 (46368404-46368533) |
| 22 | D04 | NAU3009 (48719486-48719793) |
| 23 | D09 | CIR0286 (963639-963773) |
| 23 | D09 | DPL0222 (1607197-1607428) |
| 23 | D09 | NAU3100 (1607730-1607918) |
| 23 | D09 | MUSS0300 (5438411-5438586) |
| 23 | D09 | BNL3383 (7130952-7131142) |
| 23 | D09 | TMB2527 (8389189-8389406) |
| 23 | D09 | BNL2690 (9939140-9939280) |
| 23 | D09 | NAU5325 (12115487-12115683) |
| 23 | D09 | DPL0079 (14959419-14959539) |
| 23 | D09 | MUSB0570 (21218001-21218351) |
| 23 | D09 | NAU3194 (25413614-25413778) |
| 23 | D09 | NAU2832 (28675458-28676044) |
| 23 | D09 | MUSB1040 (30479919-30480141) |
| 23 | D09 | MUSB1040 (30549848-30550064) |
| 23 | D09 | NAU4079 (30979025-30979258) |
| 23 | D09 | BNL3779 (31864392-31864595) |
| 23 | D09 | BNL3511 (32436994-32437165) |
| 23 | D09 | BNL3410 (36192913-36193134) |
| 23 | D09 | DPL0012 (39097653-39097878) |
| 23 | D09 | NAU1009 (39465566-39465758) |
| 23 | D09 | JESPR0110 (43483413-43483597) |
| 23 | D09 | NAU1047 (44634180-44634347) |
| 23 | D09 | BNL2608 (45536680-45536856) |
| 23 | D09 | BNL2590 (45746223-45746406) |
| 23 | D09 | NAU3280 (46101198-46101402) |
| 23 | D09 | HAU1683 (46320119-46320278) |
| 23 | D09 | NAU3829 (46445017-46445435) |
| 23 | D09 | DPL0395 (48340706-48340931) |
| 23 | D09 | HAU0085 (48605188-48605379) |
| 23 | D09 | MUCS006 (48696632-48696791) |
| 23 | D09 | BNL3985 (50727206-50727414) |
| 23 | D09 | DPL0541 (50924498-50924702) |
| 24 | D08 | MUSS0409 (1654324-1654526) |
| 24 | D08 | MUSS0021 (4228174-4228314) |
| 24 | D08 | BNL0252 (6984909-6985086) |
| 24 | D08 | NAU3189 (8836868-8837063) |
| 24 | D08 | NAU2829 (8920099-8920243) |
| 24 | D08 | DPL0154 (9023557-9023769) |
| 24 | D08 | DPL0111 (9528798-9529002) |
| 24 | D08 | DPL0251 (12919273-12919488) |
| 24 | D08 | DPL0755 (13365475-13365646) |
| 24 | D08 | DPL0146 (19887789-19887991) |
| 24 | D08 | TMB2919 (20039151-20039372) |
| 24 | D08 | BNL3103 (22733336-22733525) |
| 24 | D08 | NAU3632 (23594824-23595014) |
| 24 | D08 | NAU2631 (30114790-30114988) |
| 24 | D08 | HAU2522 (32316003-32316181) |
| 24 | D08 | TMB0555 (36758278-36758596) |
| 24 | D08 | JESPR0033 (37194945-37195100) |
| 24 | D08 | BNL2655 (40165867-40166020) |
| 24 | D08 | NAU5402 (44999461-44999674) |
| 24 | D08 | CIR0085 (47280907-47281140) |
| 24 | D08 | NAU0891 (49722786-49722985) |
| 24 | D08 | BNL2616 (50810313-50810460) |
| 24 | D08 | DPL0176 (53262297-53262511) |
| 24 | D08 | BNL2961 (54121768-54121988) |
| 24 | D08 | NAU3201 (56553235-56553454) |
| 24 | D08 | HAU1639 (57733162-57733312) |
| 24 | D08 | NAU4900 (57744133-57744263) |
| 24 | D08 | BNL1513 (59900879-59901015) |
| 24 | D08 | DPL0461 (60195496-60195671) |
| 24 | D08 | NAU1017 (63123021-63123175) |
| 24 | D08 | JESPR0157 (63407059-63407302) |
| 24 | D08 | BNL2772 (64300746-64300922) |
| 25 | D06 | NAU5288 (1136838-1137116) |
| 25 | D06 | HAU1481 (1235255-1235467) |
| 25 | D06 | BNL1047 (2821908-2822071) |
| 25 | D06 | HAU1355 (6119042-6119292) |
| 25 | D06 | BNL3190 (7009257-7009425) |
| 25 | D06 | JESPR0227 (14228374-14228486) |
| 25 | D06 | DPL0874 (14341574-14341786) |
| 25 | D06 | DPL0067 (18463476-18463607) |
| 25 | D06 | DPL0239 (19587878-19588120) |
| 25 | D06 | CIR0287 (21422879-21423217) |
| 25 | D06 | DPL0519 (23069365-23069578) |
| 25 | D06 | HAU1783 (25903863-25904097) |
| 25 | D06 | NAU2238 (29410870-29410997) |
| 25 | D06 | MUSB0919 (32363794-32363964) |
| 25 | D06 | HAU0668 (35496073-35496225) |
| 25 | D06 | BNL3655 (35529664-35529795) |
| 25 | D06 | NAU2838 (36761157-36761283) |
| 25 | D06 | DPL0301 (37855572-37855690) |
| 25 | D06 | BNL3103 (41309967-41310156) |
| 25 | D06 | BNL3295 (42832010-42832252) |
| 25 | D06 | NAU2565 (44066614-44066799) |
| 25 | D06 | NAU2963 (44066614-44066792) |
| 25 | D06 | DPL0290 (45111259-45111385) |
| 25 | D06 | NAU2687 (46150803-46151017) |
| 25 | D06 | CIR0299 (47619847-47619980) |
| 25 | D06 | HAU2768 (47745070-47745334) |
| 25 | D06 | NAU3524 (49243879-49244053) |
| 25 | D06 | BNL1417 (51614081-51614184) |
| 25 | D06 | NAU5373 (52781372-52781578) |
| 25 | D06 | BNL0150 (55240529-55240649) |
| 25 | D06 | NAU3171 (55711105-55711323) |
| 25 | D06 | BNL2691 (58056013-58056255) |
| 26 | D12 | HAU2826 (2655567-2655739) |
| 26 | D12 | BNL3261 (4364442-4364638) |
| 26 | D12 | NAU3897 (5642687-5642858) |
| 26 | D12 | NAU877 (6353828-6354021) |
| 26 | D12 | NAU3666 (7402337-7402547) |
| 26 | D12 | DPL0039 (8461559-8461796) |
| 26 | D12 | BNL3599 (9892165-9892355) |
| 26 | D12 | NAU2902 (10986427-10986657) |
| 26 | D12 | HAU1081 (10986440-10986623) |
| 26 | D12 | BNL0116 (13985981-13986123) |
| 26 | D12 | BNL3435 (16950703-16950837) |
| 26 | D12 | BNL3816 (17984589-17984787) |
| 26 | D12 | BNL3510 (21008334-21008469) |
| 26 | D12 | NAU3812 (21958423-21958620) |
| 26 | D12 | BNL2689 (22529459-22529589) |
| 26 | D12 | MUSS0018 (22608072-22608377) |
| 26 | D12 | DPL0183 (28286726-28286951) |
| 26 | D12 | BNL1669 (31239457-31239622) |
| 26 | D12 | DPL0280 (38368223-38368432) |
| 26 | D12 | NAU5043 (39603978-39604231) |
| 26 | D12 | DPL0770 (41753019-41753255) |
| 26 | D12 | BNL1673 (44470588-44470784) |
| 26 | D12 | BNL0341 (45881236-45881367) |
| 26 | D12 | DPL0743 (46021875-46022045) |
| 26 | D12 | NAU3236 (46196480-46196650) |
| 26 | D12 | CIR0085 (48535659-48535831) |
| 26 | D12 | BNL2495 (48929210-48929405) |
| 26 | D12 | NAU2715 (49199020-49199195) |
| 26 | D12 | NAU3662 (49966122-49966367) |
| 26 | D12 | NAU2696 (50273546-50273750) |
| 26 | D12 | BNL2725 (52951388-52951508) |
| 26 | D12 | NAU3713 (53058453-53058598) |
| 26 | D12 | NAU2640 (55981955-55982276) |
| 26 | D12 | NAU2671 (56000941-56001166) |
| 26 | D12 | NAU2030 (57802087-57802291) |

Supplementary Table 2. Summary statistics of distribution of fiber quality traits in advanced reciprocal backcross populations (BC_5_F_2_) and parental lines.

| Env | Parental means | | Acala Maxxa Background | | Pima S6 Background | |
| --- | --- | --- | --- | --- | --- | --- |
|  | Acala Maxxa | Pima S6 | Mean (Sd) | Range | Mean (Sd) | Range |
| IHF 2019 | 05.57 | 06.12 | 05.51 (0.28) | 05.00 - 06.80 | 06.51 (0.40) | 05.60 - 08.01 |
| IHF 2021 | 05.44 | 06.40 | 05.73 (0.40) | 04.70 - 06.90 | 06.53 (0.49) | 05.40 - 09.10 |
| Plains 2021 | 05.55 | 06.42 | 05.96 (0.45) | 05.10 - 07.70 | 06.65 (0.53) | 05.00 - 08.60 |

Supplementary Table 3. Summary statistics of distribution of fiber quality traits in Near-isogenic lines (NILs) and parental lines. Sd denotes sample standard deviation.

| Env | Parental means | | Acala Maxxa Background | | Pima S6 Background | |
| --- | --- | --- | --- | --- | --- | --- |
|  | Acala Maxxa | Pima S6 | Mean (Sd) | Range | Mean (Sd) | Range |
| IHF 2021 | 05.44 | 06.40 | 05.80 (0.48) | 04.50 - 07.80 | 6.39 (0.52) | 04.70 - 08.40 |
| Plains 2021 | 05.55 | 06.42 | 05.82 (0.46) | 04.60 - 08.70 | 6.56 (0.54) | 05.00 - 08.50 |

Supplementary Table 4. Analysis of variance of fiber quality traits. DF denote degrees of freedom, SS denotes sum of squares, MS denotes mean sum of squares, PVE denotes percent variance explained by the factor. ABL = Advanced backcross lines, NIL = Near-isogenic lines.

| Pop |  | Acala Maxxa background | | | | | Pima S6 background | | | | |
| --- | --- | --- | --- | --- | --- | --- | --- | --- | --- | --- | --- |
|  | Source | DF | SS | MS | F-value | PVE | DF | SS | MS | F-value | PVE |
| ABL | ENV | 2 | 26.53 | 13.27 | 142.48*** | 18.10 | 2 | 5.40 | 2.70 | 22.07*** | 2.14 |
|  | REP(ENV) | 3 | 0.15 | 0.05 | 0.53 | 0.10 | 3 | 3.17 | 1.06 | 8.64*** | 1.25 |
|  | GEN | 172 | 53.62 | 0.31 | 3.35*** | 36.59 | 230 | 110.30 | 0.48 | 3.92*** | 43.62 |
|  | GEN*ENV | 287 | 31.90 | 0.11 | 1.19 | 21.77 | 410 | 76.24 | 0.19 | 1.52*** | 30.15 |
|  | Error | 369 | 34.36 | 0.09 |  | 23.44 | 472 | 57.73 | 0.12 |  | 22.83 |
| NIL | ENV | 1 | 0.00 | 0.00 | 0.00 | 0.00 | 1 | 10.8 | 10.8 | 67.17*** | 2.89 |
|  | REP(ENV) | 2 | 0.42 | 0.21 | 1.81 | 0.13 | 2 | 2.63 | 1.31 | 8.17*** | 0.70 |
|  | GEN | 397 | 207.27 | 0.52 | 4.51*** | 63.83 | 407 | 223.19 | 0.55 | 3.41*** | 59.80 |
|  | GEN*ENV | 389 | 40.32 | 0.10 | 0.90 | 12.42 | 369 | 52.53 | 0.14 | 0.89 | 14.07 |
|  | Error | 663 | 76.71 | 0.12 |  | 23.62 | 523 | 84.09 | 0.16 |  | 22.53 |
